# Anisotropic functionalized platelets: percolation, porosity and network properties†

**DOI:** 10.1039/d3na00621b

**Published:** 2023-12-11

**Authors:** Carina Karner, Emanuela Bianchi

**Affiliations:** a Institut für Theoretische Physik, TU Wien Wiedner Hauptstraße 8-10 A-1040 Wien Austria carina.karner@tuwien.ac.at emanuela.bianchi@tuwien.ac.at; b CNR-ISC, Uos Sapienza Piazzale A. Moro 2 00185 Roma Italy

## Abstract

Anisotropic functionalized platelets are able to model the assembly behaviour of molecular systems in two dimensions thanks to the unique combination of steric and bonding constraints. The assembly scenarios can vary from open to close-packed crystals, finite clusters and chains, according to the features of the imposed constraints. In this work, we focus on the assembly of equilibrium networks. These networks can be seen as disordered, porous monolayers and can be of interest for instance in nano-filtration and optical applications. We investigate the formation and properties of two dimensional networks from shape anisotropic colloids functionalized with four patches. We characterize the connectivity properties, the typical local bonding motives, as well as the geometric features of the emerging networks for a large variety of different systems. Our results show that networks of shape anisotropic colloids assemble into highly versatile network topologies, that may be utilized for applications at the nanoscale.

## Abstract 2

Anisotropic functionalized platelets are able to model the assembly behaviour of molecular systems in two-dimensions thanks to the unique combination of steric and bonding constraints.

## Graphical abstract

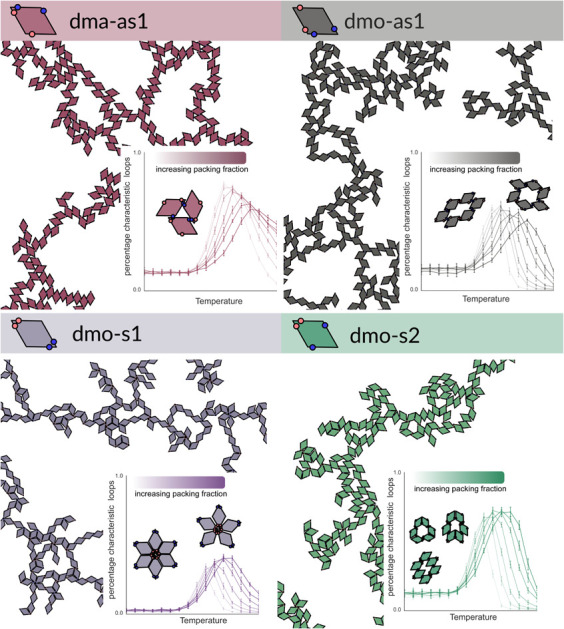

## 1 Introduction

Highly ordered monolayers with pores at the nano-scale are heavily sought after as their electronic, optical and mechanical properties can be taken advantage of in many applications ranging from sensing to catalysis (as chemical reactions can be confined to the nanopores and thus easily tracked), electron or mass transport, selective adsorption (as functionalized pores can selectively bind to specific molecules) or gas separation and storage.^2–7^ The fabrication of ordered porous structures has accelerated thanks to development of new materials such as metal–organic frameworks (MOFs), covalent organic frameworks (COFs), hydrogen-bonded organic frameworks (HOFs), and amorphous materials such as amorphous silica and amorphous carbon.^8–15^ These are a wide variety of different systems: MOFs are compounds consisting of metal ions or clusters connected to organic ligands by coordination bonds, COFs are building blocks featuring light elements (such as C, H, O, N, or B atoms) connected through covalent bonds, HOFs are building blocks featuring light elements (such as C, H, O, N, or B atoms) connected through hydrogen bonds, amorphous silica is silicon dioxide (SiO_2_) that does not form any crystalline structure in two dimensions but rather extended disordered networks, similarly to amorphous carbon where the structure of the network is governed by the hybridization status of the atoms. Moreover, the formation of two dimensional patterns in these classes of systems can sometimes be influenced by the solvent, the presence of a substrate or of possible precursors.^16–21^ Despite the broad differences between specific systems, the core of their assembly strategy is the same and is based on taking advantage of – or even designing – molecular components such that specific bonding patterns are enforced which are characterized by the presence of predictable voids. Even if the building blocks and their mutual interactions can vary extensively, they all have two features in common: bond directionality and limited bonding valence.

While ordered porous frameworks are established to be relevant for applications in a wide range of fields, covering engineering, physics, chemistry, biology and medicine, the impact of disordered states including “defective by design” crystals, as well as amorphous phases such as glasses and gels is still to be understood.^22–26^ “Defective by design” open crystals are space filling networks with a long-range order, where the arrangement of the nanopores has a controllable degree of disorder.^25,26^ In contrast, glasses and gels are disordered phases characterized by diffuse scattering patterns due to the absence of long-range order^22–24,27^ and emerge, *e.g.*, under rapid cooling, where a careful choice of the annealing conditions can tune the porosity of the assembled network.

Given the broad spectrum of atomic and molecular units forming ordered and disordered assemblies, numerical tools offer systematic approaches to the understanding as well as the design of these systems. While DFT calculation and full monomer simulations focus on the microscopic details,^28–32^ such as possible energy differences between different bonding arrangements, numerical simulations of appropriately designed coarse-grained models can be used to investigate the rationale behind the emergence of specific patterns with respect to others.^33–40^ In the present contribution, we aim at understanding how the intrinsic shape anisotropy of atoms and molecules and the anisotropy of their specific bonding patterns, together, affect the formation and the features of disordered networks. In particular, we focus on how the physical properties of the networks – such as pore sizes and shapes – change by varying the particle bonding patterns. Here, we investigate the formation of two dimensional networks in systems of elongated, functionalized molecules by considering rhombic platelets arbitrarily decorated with bonding sites – referred to as patches – along their edges. This coarse-graining approach was first introduced to understand the assembly of tetracarboxylic acids on a graphene substrate:^33^ the molecules were modeled as regular hard rhombi (with interior angles 60° and 120°) decorated with four bonding patches, each placed at the center of a platelet edge. The presence and position of the bonding sites, together with the steric incompatibilities of the hard shapes, were shown to steer the assembly towards the same tiling observed in the experimental molecular systems.^41^

In our previous work,^1^ we further extended the model parameter space by introducing different patch arrangements along the particle's perimeter – corresponding to, *e.g.*, different molecules – as well as attractive and repulsive patches – due to *e.g.* different deprotonation levels or different functional groups. Different patch arrangements already emerge when considering different tectons of tetracarboxylic acids,^29,42,43^ where the elongation of the molecule's backbone implies a different relative position of the bonding groups. While these reference molecules have four equal functional groups, molecules with distinct interaction sites are within experimental reach. In fact, even though the rational design of molecules with more than one bonding type is still in its infancy, different interaction sites might emerge either due to different deprotonation levels of the molecules or thanks to the presence of different functional groups. The extent of deprotonation of organic carboxylate ligands – easily controlled by the solvent, the pH or the temperature^44^ – can for instance influence the ability of the tetracarboxylic acids to coordinate with metal ions^45^ as well as the binding motifs between the tetracarboxylic molecules themselves.^46,47^ Moreover, orthogonal synthesis techniques for dynamic covalent reactions are recently emerging as a tool to control the chemical specificity of organic molecules^48,49^ and a few molecular tilings originating from this technique have been observed.^50^ In order to span the board variety of resulting patch arrangements, we created sets of symmetry operations and patch movements that systematically sample a large subset of possibilities. By investigating the assembly of this kind of systems, we have shown that,^1^ changes in the patch positions and patch identity can lead to very diverse tiling scenarios. Most of the studied systems formed space-filling monolayers, either close-packed or porous, where all particles were fully bonded. The emergence of a specific tiling was characterized in terms of steric incompatibilities and bond specificity, while the porosity was linked to the way patches were placed off-center: the more off-center the patches are placed, the larger the pores become, while the lattice ordering is preserved. A small subset of systems was observed not to form any tilings. In the present work we target those particle types and systematically investigate their assembly. We show that these systems form disordered extended networks of bonded particles, whose particle loops/pores depend on the interplay between shape and bond anisotropy.

## 2 Model and methods

**Fig. 1:**
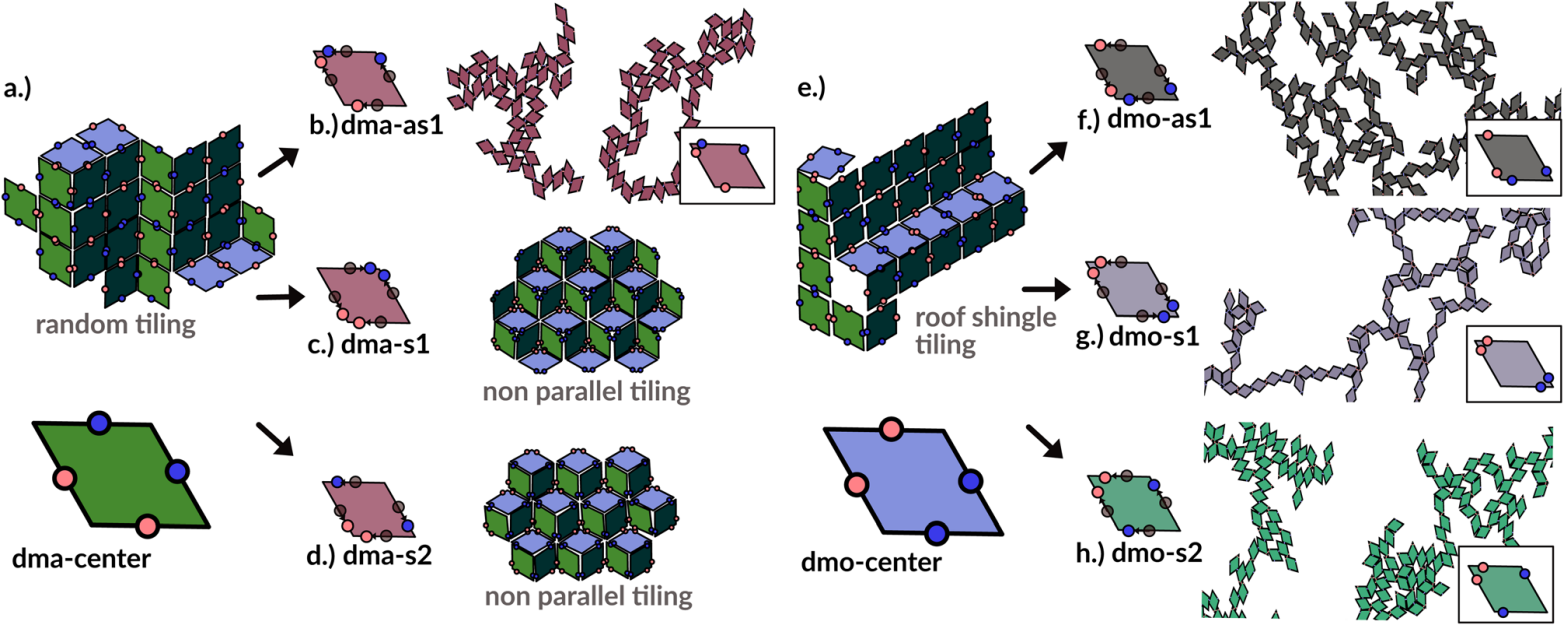
Selection of assembled structures of regular patchy rhombi with four patches of two kinds (red and blue spots): same-type patches are mutually attractive, while different-type patches are mutually repulsive; three different particle types are displayed, classified by where the same-type patches sit. In (a), (c)–(e) particles in assembly sketches are colored according to the particle orientation to highlight the different assembly patterns. All space-filling tilings have been studied in ref. 1 and are reported here for a complete overview, while disordered networks are studied in the present work. First column (a–d): double-manta-systems (dma): same-type patches enclose the large rhombus angles; (a) double-manta-center (dma-center) particle type, where all patches sit in the center of the edges, assembles into a random tiling;^1^ (b) double-manta-asymmetric-1 (dma-as1), where same-type patches are shifted off-center asymmetrically, results in a network structure and are studied in the present work; (c) double-manta-symmetric-1 (dma-s1) and (d) double-manta-symmetric-2, where same-type patches are shifted off-center symmetrically, result in non-parallel tilings.^1^ Second column (e–g): double-mouse-systems (dmo): same-type patches enclose small rhombus angles; (e) double-mouse-center (dmo-center), where all same-type patches sit in the center of the edges, organizes into a roof-shingle tiling (note that the roof-shingle tiling differs from the random tiling as the characteristic motives of the latter – three particles joint together by non-parallel bonds – rarely appear in favor of parallel and zigzag bond motives);^1^ (f) double-mouse-asymmetric-1 (dmo-as1), where same-type patches are shifted off-center asymmetrically, forms a network and is subject of the present study; (g) double-mouse-symmetric-1 (dmo-s1) and (h) double-mouse-symmetric-2 (dmo-s2), where same-type patches are shifted off-center symmetrically, organize into disordered networks and are observed in the present study.

**Fig. 2:**
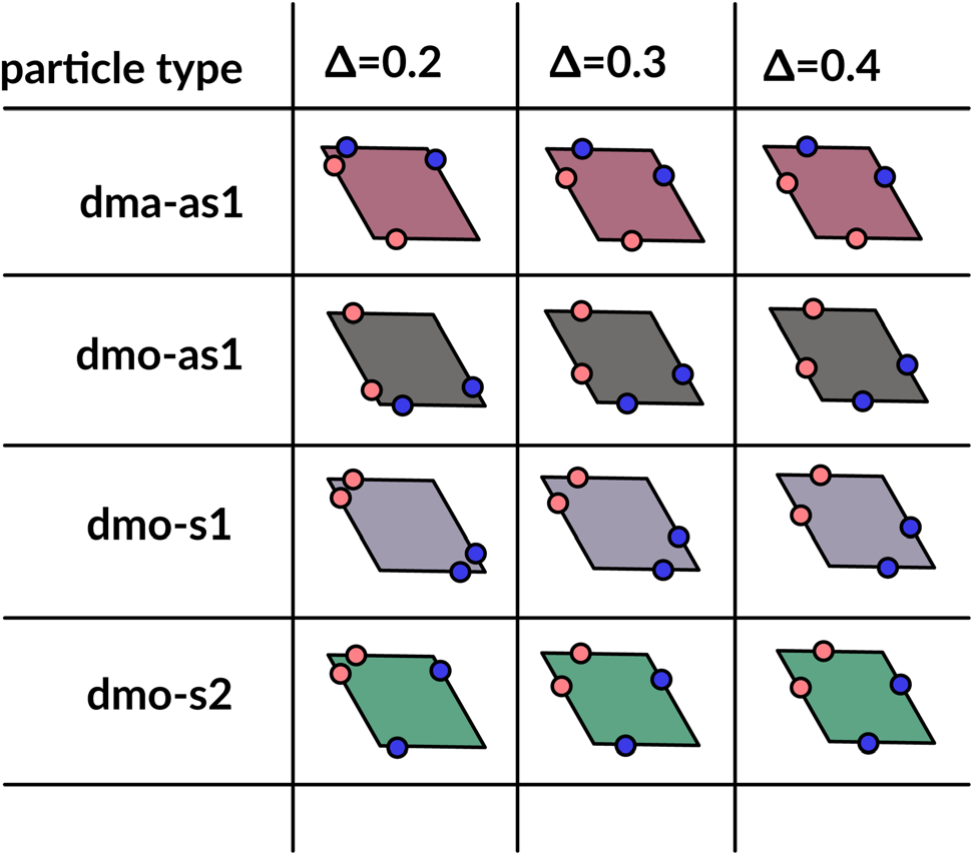
Graphical summary of all studied particles types – dma-as1, dmo-as1, dmo-s1 and dmo-s2 – and patch positions *Δ* = 0.2, 0.3, 0.4.

In order to systematically span the model parameter space, we classified^1^ patchy rhombi with four patches of two different kinds according to their patch arrangement, given in turn by a patch topology and a patch placement. The patch topology defines the pattern of patches of the two types, while the patch placement sets the exact patch position along the rhombi edges. For rhombi with two patch types we introduced two topologies: when patches of the same kind enclose the larger rhombus angles, the topology is referred to as double-manta (dma) and when patches of the same kind enclose the smaller rhombus angles, the topology is referred to as double-mouse (dmo). The specific patch arrangement for a given topology should be described by a set of four distances measuring the position of each patch with respect to its reference vertex. Nonetheless, as we introduced symmetry operations on the patch arrangement, we can describe each particle type by one single parameter, *Δ*, for a given patch topology. By systematically exploring the systems parameter space, we have shown that^1^ the assembly of these different systems can be tuned by changes in the patch positions and patch identity, leading to very diverse tiling scenarios (Fig. 1). In a small subset of systems – reported in Fig. 2 – the emergence of a tiling was not observed. In this study, we focus on them and conduct a methodical investigation into their assembly process.

**Fig. 3:**
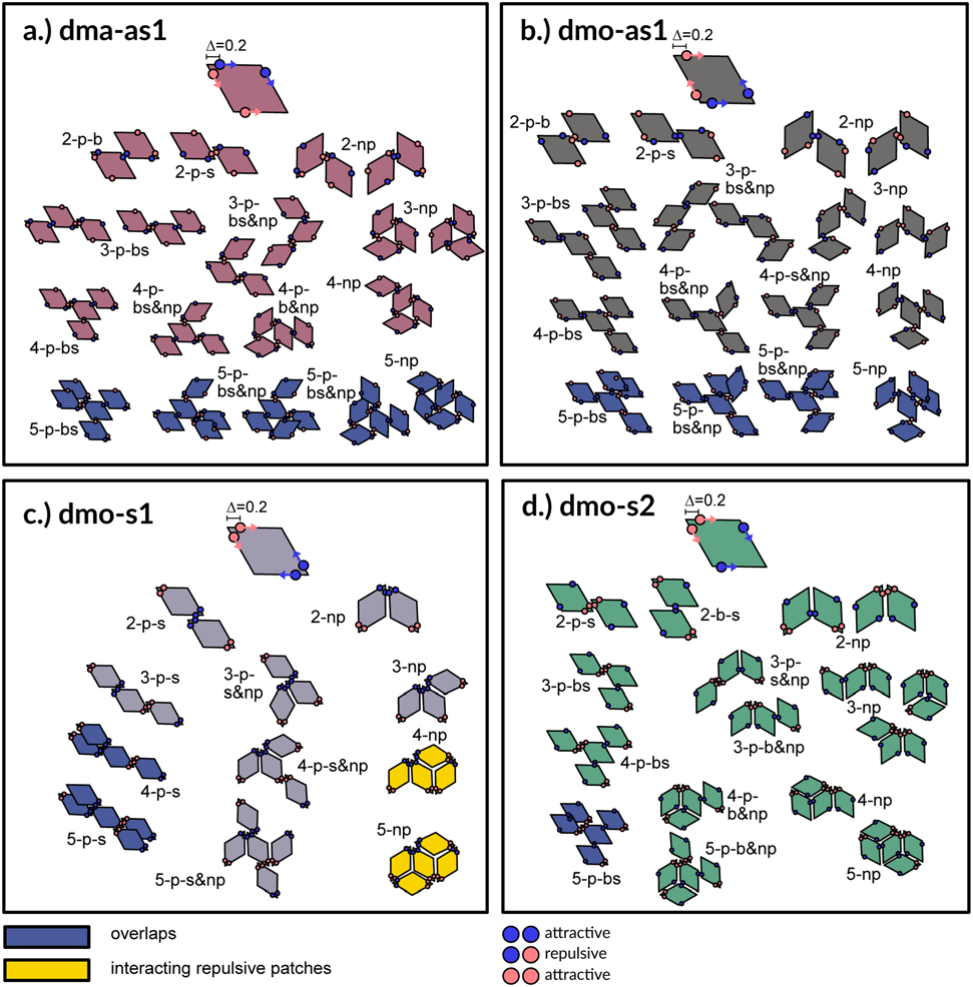
Particle types and small cluster analysis. The small-cluster-analysis for the four studied particle types with *Δ* = 0.2 shows a selection of available bonded arrangements, from clusters of two up to five particles: sketches are built up such that particles are added one at a time from top to bottom to the depicted small clusters. While forbidden or disfavoured clusters are colored in blue and yellow, respectively, clusters without overlaps and with energetically favorable bonds are colored in the respective color of the particle type used throughout the paper: dma-as1 is colored in burgundy, dmo-as1 in grey, dmo-s1 lilac, dmo-s2 sea-green. The different clusters are named according to a scheme. All variable names: parallel (p), non-parallel (np), parallel bonds enclosing particle's big angles (b), parallel bonds enclosing particle's small angles (s), parallel bonds enclosing small angle as well as big angles within a cluster (bs). Exemplary, 3-p-s & np corresponds to a cluster of three particles with mixed parallel and non-parallel bonds, where the patches bonded in a parallel fashion enclose the particle small angles.

We consider four different particle types: dma-as1, dmo-as1, dmo-s1, dmo-s2 – as depicted in Fig. 3 for a given *Δ*-value. Note that *Δ* generally can take values between 0 and 1, it denotes the distance of one patch from its reference corner and, together with the selected patch topology, it fully defines the positioning of the other three patches. In symmetric patch topologies (labelled s for symmetric), patches of the same kind are placed symmetrically with respect to their shared vertex; dmo-s1 and dmo-s2 differ in how the patches move in concert with changes in *Δ*: in the s1 case, both pairs of patches of the same kind move towards their shared vertex on decreasing *Δ*; in the s2 case, on decreasing *Δ*, one pair of patches of the same kind moves towards the shared vertex, while the other pair moves away from it. In asymmetric patch topologies (labelled as for asymmetric), pairs of patches of the same kind – either in the dma or in the dmo pattern – are described by {*Δ*, 1 − *Δ*}; in the case of interest here (labeled as1) different patches on adjacent edges move either closer or further way on increasing *Δ*. It is worth noting that the nomenclature of the particle types is chosen to be consistent with our previous work.^1^ We consider three values of *Δ* = 0.2, 0.3 and 0.4 per topology.

The interaction between a pair of rhombi *i* and *j* is given by
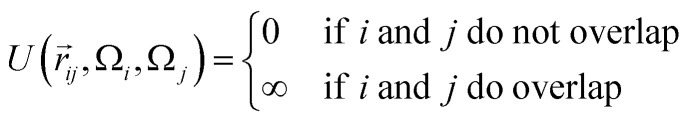
where *r⃑*_*ij*_ is the center-to-center distance vector, and *Ω*_*i*_ and *Ω*_*j*_ describe the particle orientations. As our hard rhombi are convex polygons, we use the separating axis theorem^51^ to detect whether two of them overlap or not. We set the side length of the rhombi *l*_r_ to 1. The interaction between pairs of patches is defined by an attractive or repulsive square-well potential:
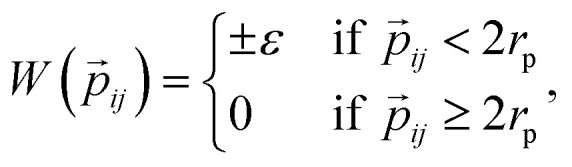
where *p⃑*_*ij*_ is the patch-to-patch distance, 2*r*_p_ is the patch diameter and *ε* denotes the patch interaction strength. We set *r*_p_ = 0.05 – as this condition guarantees that each patch can at maximum bond to another patch^52^ – and *ε* = ±1. Patches of the same kind attract each other, while patches of a different kind repel each other, thus an interaction between attractive patches results in a bond, while the interaction between repulsive patches does not. The Monte Carlo-evaluation of the total energy consists of a collision detection check – where the particle orientation/translation move is accepted if the selected particle does not overlap to any other particle – and the subsequent calculation of the patch interaction energy.

We run Monte Carlo (MC) simulations in two dimensions in the canonical ensemble with single particle rotation and translation moves as well as cluster moves.^53,54^ We fix the number of particles to *N* = 1500 and build a grid of investigated state points: we consider *m* = 16 temperatures *T*, with *T*_0_ = 0.01 and *T*_m_ = 0.16, and *n* = 24 packing fractions *ϕ*, with *ϕ*_0_ = 0.01 and *ϕ*_*n*_ = 0.525 which amounts to a total of 384 state points per system (see Table 1 for a summary of all selected particle and simulation parameters). We note that our previous investigations on the same systems were carried out in the grand-canonical ensemble at fixed interaction energy – corresponding to *T* ≈ 0.019 for systems where *ε* was set to the unity – and at a fixed chemical potential – leading to an average packing fraction of about 0.1 (corresponding to an equilibrium gaseous phase). We follow the same simulation protocol for all investigated systems: for each state point we start from a square lattice at the given packing fraction and we equilibrate the system by switching off the patch interaction for 1 × 10^6^ MC-sweeps, which is equivalent to simulating the systems at very high temperatures, then we switch on the patches and quench these systems for 2–3 × 10^7^ MC-sweeps to the given temperatures and collect statistics for the subsequent 1 × 10^6^ MC-sweeps. For each state point of each system, we perform 16 equivalent runs. Considering all parallel runs and all 384 states across all particle topologies and *Δ*-values, in total we conducted 73 728 separate simulations.

**Table 1:** Table of used simulation and particle parameters

Simulation parameters		
Name	Variable	Range/amount
Number of particles	*N*	1500
Packing fraction	*ϕ*	0.01–0.525
Temperature	*T*	0.01–0.16
Number of parallel runs	—	16
Particle type	dma-as1, dmo-as1, dmo-s1, dmo-s2	4
Patch position	*Δ*	0.2, 0.3, 0.4
Patch interaction energy	*ε*	±1.0
Rhombus side length	*l*_r_	1.0
Rhombus small angle	*α*	60°

## 3 Results

### Small cluster analysis

A first qualitative insight of the available assembly avenues for the selected systems can be derived by the analysis of the local bonding patterns. For each particle type, we report a (not necessarily exhaustive) set of possible small clusters, from size two up to five (see Fig. 3), built up step by step with the aim of satisfying the highest number of bonds: we consider either only parallel (p) or non-parallel (np) bonds as well as mixed bonding patterns (p & np). According to this small cluster analysis – that is independent of temperature and packing fraction, and just takes into account local bonding possibilities – we may already infer that fully bonded crystals are hardly realized or not achievable at all, because of either steric incompatibilities or energetic penalties. More specifically, in dma-as1 and dmo-as1 systems, the growth possibilities of a small cluster towards a fully bonded pattern are strongly limited by steric incompatibilities. In particular, in dma-as1 systems, candidate building blocks of a non-parallel tiling, previously labelled as open boxes, cannot tile into a fully bonded pattern because of particle–particle overlaps (see clusters 3-np *versus* 5-np in panel (a) of Fig. 3); the same is true for candidate building blocks of a parallel tiling (see cluster 4-p-bs *versus* 5-p-bs in the same panel). Similar considerations can be issued for dmo-as1, where bonding a fifth rhombus to a pre-bonded cluster of size four necessarily results into an overlap (see all sample clusters of size five reported in blue at the bottom of panel (b)). In dmo-s1 systems, the parallel tiling is prevented by particle–particle overlaps (see 3-p-s *versus* 4-p-s and 5-p-s in panel (c)), while the non-parallel tiling is possible but energetically disfavored (see 4-np and 5-np in panel (c)) as mutually repulsive patches would interact. Growth directions where parallel and non-parallel bonds are mixed involve either overlaps or energy penalties (see, *e.g.*, cluster 5-p-s & np in panel (c)). Finally, in dmo-s2 systems, parallel growth is prevented by overlaps (see cluster 5-p-bs in panel (d)), while non-parallel tiling is allowed (see *e.g.*, cluster 5-np in the same panel). Nonetheless, even though a rhombus can be fully bonded within such a non-parallel tiling, its neighbors cannot achieve the same status (see both clusters 5-np and 5-p-b & np), therefore, also in this case, four average bonds per particle are never achieved. In summary, it is reasonable to assume that fully bonded crystals are not achievable for these four particle types, instead we expect assembly scenarios characterized by two to three bonds per particles on average, opening up the possibility to form states, without positional and orientational order.

### Network analysis

**Fig. 4:**
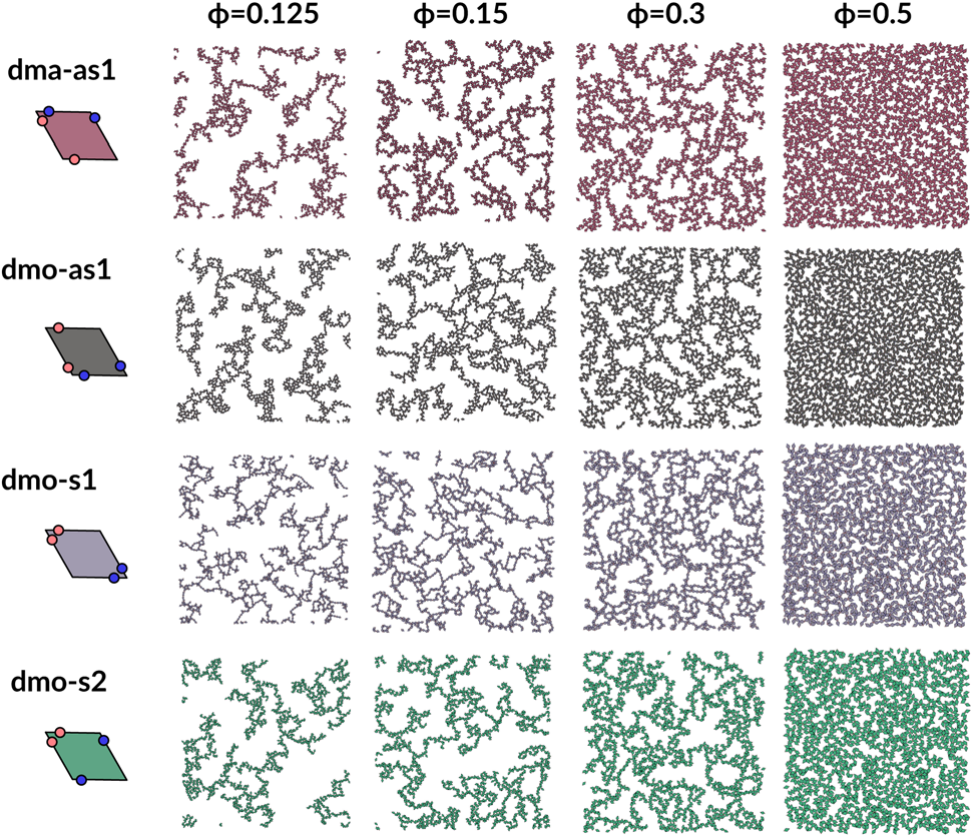
System snapshots at the lowest investigated temperature *T* = 0.01 and four different packing fractions *ϕ* = [0.125, 0.15, 0.3, 0.5], as labeled, for systems with *Δ* = 0.2, from top to bottom: dma-as1 (burgundy), dmo-as1 (grey), dmo-s1 (lilac), dmo-s2 (sea-green), as labelled.

**Fig. 5:**
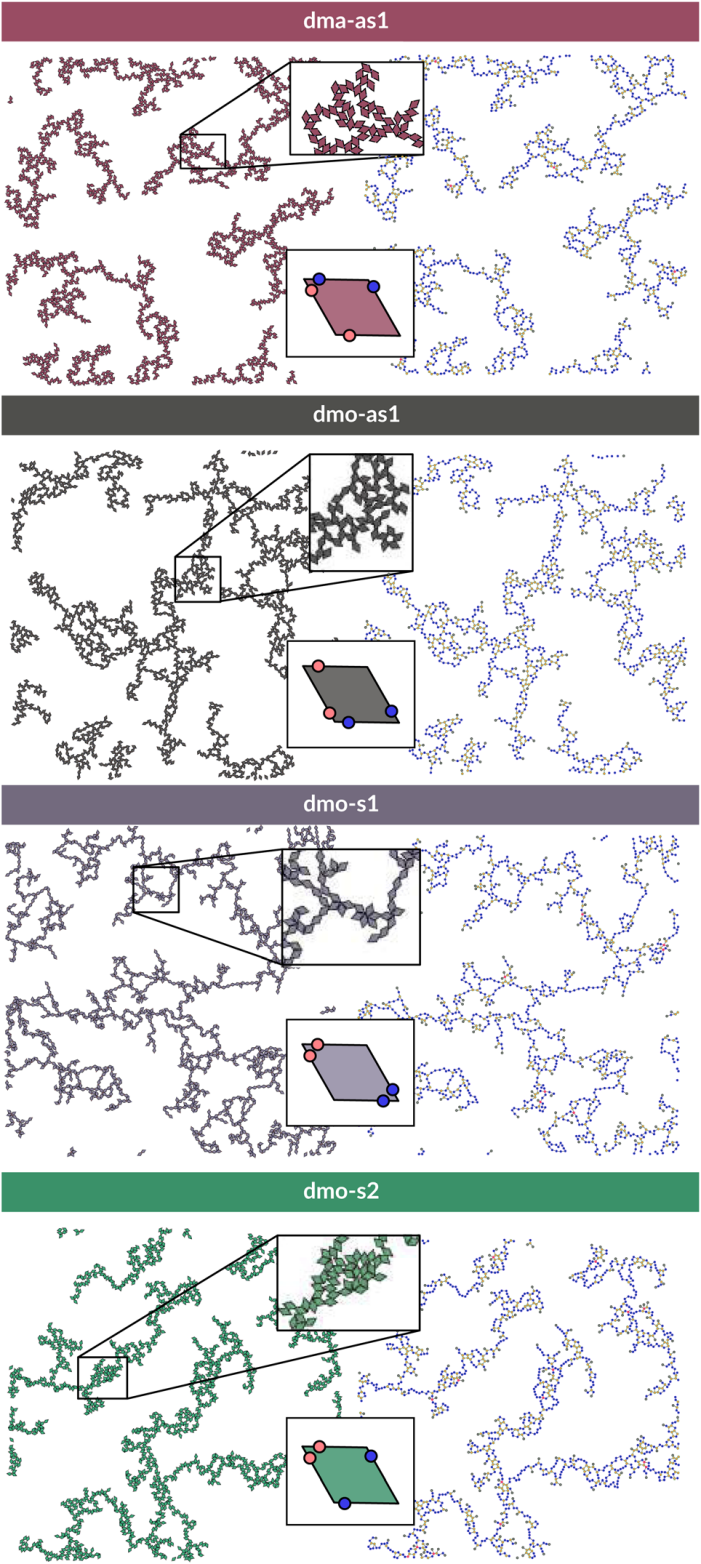
Left column: system configurations at temperature *T* = 0.01, packing fraction *ϕ* = 0.125 and patch position *Δ* = 0.2; right column: corresponding network representations, where the particle centers are drawn as circles (nodes) and connections are drawn between bonded neighbours. Periodic boundaries still apply. Systems from top to bottom: dma-as1, dmo-as1, dmo-s1 and dmo-s2, as labelled. In the network representation particles are represented by nodes (colored circles) and bonds are represented by the connecting edges (lines), coloring is according to the node degree – the number bonded neighbours: nodes of degree one (gray), nodes of degree two (blue), nodes of degree three (yellow), nodes of the degree four (red).

To investigate what happens at different thermodynamic conditions we consider a large set of state points. A first scan of the assembly behaviors can be performed on increasing the packing fraction at the lowest temperature studied, *i.e.*, at *T* = 0.01 (see selected snapshots reported in Fig. 4 for particle topologies with *Δ* = 0.2). At such a low temperature, bonding between particles is highly favored and thus an extended cluster of bonded particles emerges that connects the majority of the particles and spans the whole system from side to side. The resulting networks differ already at a simple visual inspection. This is particularly true at *ϕ* = 0.125 and *ϕ* = 0.15 where a few branched-out backbones (branches) appear to conjoin and thus connect the whole network, while on increasing the packing fraction towards *ϕ* = 0.5 all systems become more homogeneous. At low packing fractions, instead, the systems are characterized by large voids and relatively small pores, where both the branch thickness and the pore features of the branches differ visually according to the particle type. A zoom-in of the lowest packing fraction snapshots is reported in Fig. 5 where the effect of the characteristic bonding motifs of the different particle types can be appreciated. The visual inspection suggests that, dmo-s1 particles form the thinnest network branches, while dmo-s2, dma-as1 and dmo-as1 are characterized by thicker ones: these latter three systems have smaller pores, while dmo-s1 has larger and less regular pores. It is worth noting that we do not rank the thickness of the branches as this is a non-trivial task with two aspects to consider: while the spatial thickness of a branch – defined as its average linear dimension along a given direction – seems very similar for the three systems with thicker branches, the particle thickness/branch density – defined by the average number of particles along the same given direction – appears to be quite different (see the zoomed-in insets in Fig. 5), and in this respect dmo-s2 may display a higher branch density compared to dma-as1 and dmo-as1. Clearly, the thickness of the branches consistently increases on increasing *Δ* for all systems, as the bonding becomes less off edge and more on-edge.^1^

The features of the network can be better understood, at least at a qualitative level, by taking advantage of the small cluster analysis. First of all, it is worth noting the difference between parallel bonds enclosing small angles (p-s) and parallel bonds enclosing big angles (p-b): for a given *Δ*-value, p-s bonds establish a larger distance between the particle centers with respect to the characteristic inter-particle distance guaranteed by the p-b bonds (see for instance clusters 2-p-s and 2-p-b in panel (b) of Fig. 3). While in dmo-s2, dma-as1 and dmo-as1 systems both bond types are possible, in dmo-s1 only p-s bonds are allowed, thus resulting in more stretched network branches compared to the other three systems. Further, the morphology of the different networks can be qualitatively described in terms of the local bonding motifs. In particular, in dma-as1 networks, open boxes and zig-zag chains are observed and can be directly related respectively to 3-np and p-bs bonding motives, while kinks are introduced by off-edge particle bonds in np motives. In dmo-as1 networks, the 3-np clusters do not form closed loops – thus giving rise to slightly less compact assemblies – while p-bs and np motives still guarantee zig-zags and kinks. In dmo-s1 assemblies, np motifs lead to an on-edge particles arrangement and thus no kinks are observed. Finally, in dmo-s2 aggregates, zig-zag chains introduced by p-bs are observed, while np motives occur on-edge thus leading to the formation of clusters such as close-packed (not-fully bonded) boxes (see the 3-np clusters for this system).

In the following we aim at characterizing the network features as well as at identifying the locus of state points where a system-spanning network of particles first emerges.

To characterize the branching of the networks we use graph theory, where we analyse the forming network only based on connectivity and ignore the physical aspects of the aggregated particles, *i.e.*, the particle positions, orientations and bonding angles. Thus, the particle network is treated as an abstract network, where each particle becomes a network node and each bond between two particles becomes a network edge connecting two nodes. At this point it is important to note that all network properties discussed in this paper are obtained by our own software^55,56^ which relies heavily on Networkx,^57^ a Python module for drawing and analysing graph data. We compare simulation snapshots of the particle networks at *T* = 0.01, *ϕ* = 0.125 and *Δ* = 0.2 (see Fig. 5, left column) with a graph representation, where the particle centers are drawn as circles: they represent the nodes of the graph and are connected to their bonded neighbours by lines. By omitting the orientations of the particles and the geometry of their patch connection, focus is drawn to the most basic network features. The most basic measure for characterizing graphs is the node degree, which is the number of connecting edges of each node. In the depicted networks, nodes are colored according to their degree, and already by inspecting the coloring it becomes visually apparent that, despite of differences in their physical features, the occurrences of chaining elements – nodes of degree two (blue) – and branching elements – nodes of degree three (yellow) – are very similar across all investigated systems.

**Fig. 6:**
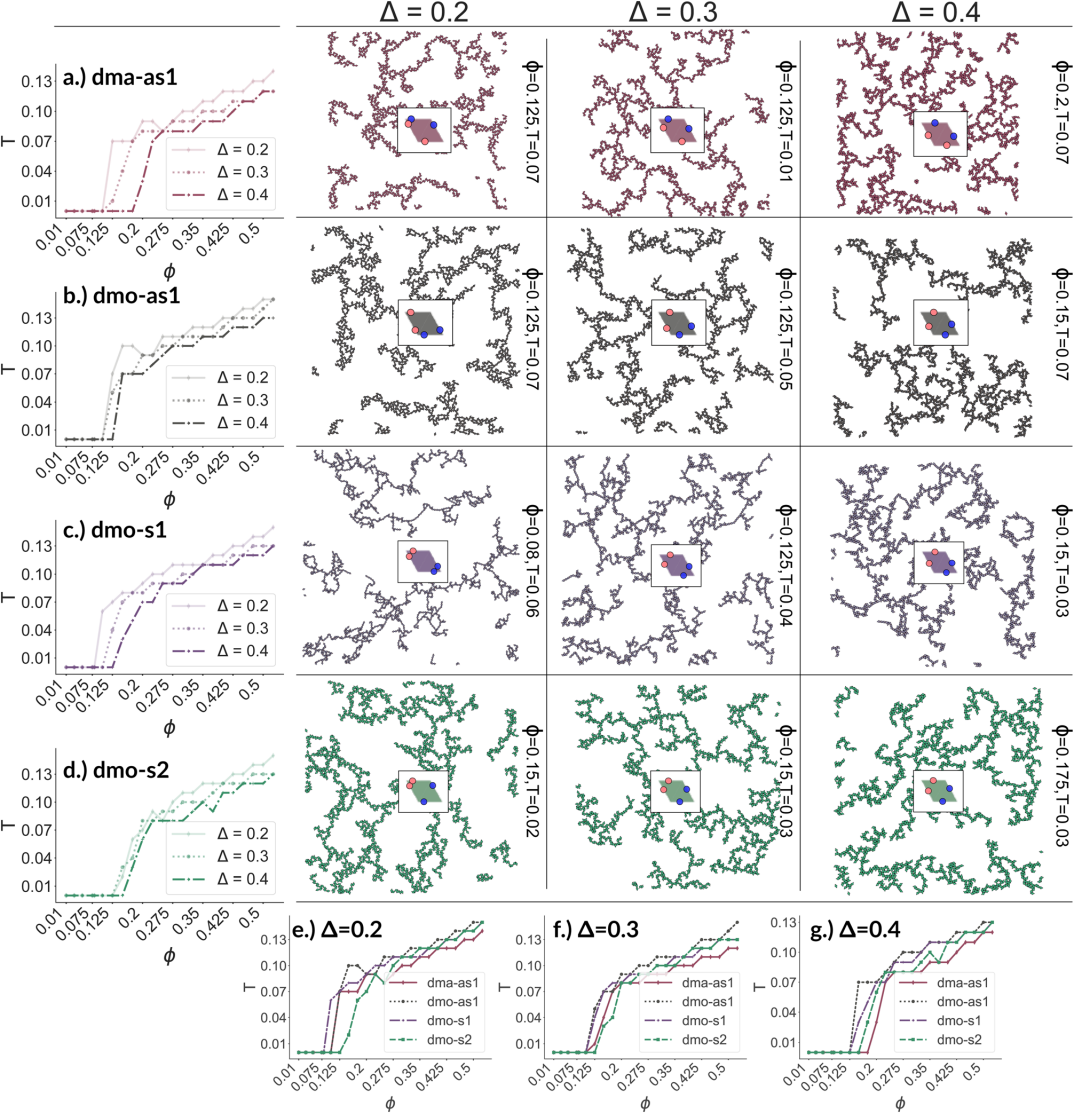
Panels (a)–(d), first column: percolation lines per particle type. Here, the percolation line is drawn as function of the packing fraction: each point corresponds to the highest temperature at which at least 50% of all parallel runs of each system are percolated for that given packing fraction. From top to bottom: (a) dma-as1, (b) dmo-as1, (c) dmo-s1 and (d) dmo-s2, as labelled. Second to fourth column: simulation snapshots of configurations at the lowest packing fraction where percolation was observed for each particle type, from left to right *Δ* = [0.2, 0.3, 0.4]; corresponding temperature *T* and packing fraction *ϕ* are denoted at the right of each snapshot. Panels (e–g): percolation lines per patch position *Δ*, from left to right: *Δ* = [0.2, 0.3, 0.4].

We now broaden our perspective to the whole grid of investigated state points and characterize the percolation line, *i.e.*, the locus of state points where a system-spanning (percolating) network of bonded particles first appears. We define a system to be percolating when half of the parallel runs exhibit a cluster that extends beyond the periodic simulation box to infinity in at least one direction.^58^ This condition allows to properly distinguish between an extended network and a compact single crystallite. In Fig. 6 (panels (a)–(d), first column) we report the percolation lines in the *T versus ϕ* plane, each panel refers to a different particle type and for each particle type we report all the three *Δ*-values investigated. As expected, the percolation temperature is monotonously increasing with increasing packing fraction. For all particle types and patch positions, the percolation lines are within a similar range of temperatures and packing fractions. The lowest packing fraction where percolation was observed is between *ϕ* = 0.10–0.175 for a temperature range between *T* = 0.05 and *T* = 0.1. Snapshots in Fig. 6 (second to fourth column, four panels each from top to bottom) show configurations at these particular percolation temperatures and packing fractions for each particle type and *Δ*. Below these state points the packing fraction is too low for the particles to span the whole system. At the highest packing fraction investigated, *ϕ* = 0.525, the percolation temperature is between *T* = 0.12 and *T* = 0.15. On increasing *Δ*, the percolation line consistently moves towards lower temperatures for each particle types, meaning that, for a given *ϕ*, more symmetric patch configurations percolate at lower temperatures for all particle types. The robustness of this behavior in *Δ* is confirmed by comparing panels (e)–(g) of Fig. 6, where we report the percolation lines in the *T versus ϕ* plane grouped by *Δ*-values. It is interesting to note that for dmo-s1 systems, that visibly show the thinnest branches, the onset of the percolation consistently occurs at lower densities compared to the other systems at all *Δ*-values. This may be understood by acknowledging that thin branches span a larger fraction of the sample with the same amount of particles of a thick branch.

The similarity between the percolation scenarios of the different particle types can be rationalized in view of the striking similarities between the different networks, already discussed in Fig. 5: all networks are constituted by chain elements of degree two, *i.e.* particles forming two bonds, and branch elements of degree three, *i.e.* particles with three bonds.

**Fig. 7:**
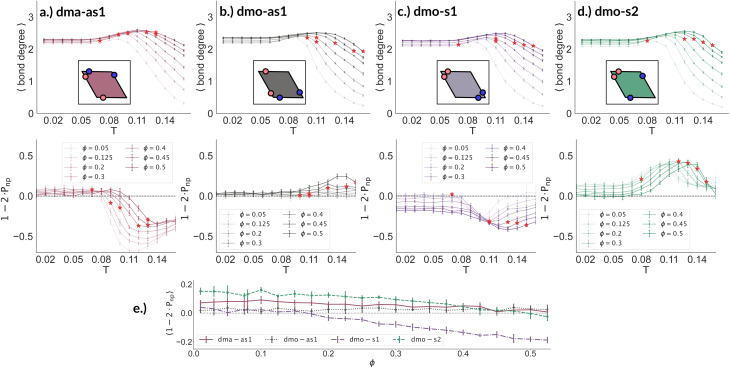
Average bond degree (first row) and bond orientation order parameter 1 − 2 × *P*_np_ (second row), each as function of temperature *T*, at different packing fractions *ϕ* = [0.05, 0.125, 0.2, 0.3, 0.4, 0.45, 0.5] for particle topologies with patch position *Δ* = 0.2: top–bottom panels (a) dma-as1 (burgundy), (b) dmo-as1 (grey), (c) dmo-s1 (lilac) and (d) dmo-s2 (sea-green), as labelled. Red stars highlight the state point at which percolation occurs. Third row, panel (e): bond order parameter 1 − 2 × *P*_np_ of all particle types as function of *ϕ* for *T* = 0.01 and *Δ* = 0.2.

To better understand the percolation scenario we determine the average number of bonds per particle. In contrast to the small cluster analysis, the average number of bonds per particle, or, in network terminology the average bond degree, takes into account both the local bonding possibilities and the thermodynamic conditions: it thus corresponds to the effective functionality of our rhombic platelets at a given state point. In Fig. 7 (panels (a)–(d), first row) we plot the average number of bonds for each system with *Δ* = 0.2 as a function of *T* for different *ϕ*-values. We observe that the average bond degree is between zero and two in the fluid phase that appears at high temperatures, it is between two and three in the vicinity of the percolation, while well below the percolation it becomes close to two. The fully bonded scenario, corresponding to an average bond degree close to four, is never observed for any particle type at any *Δ*-value (see also the ESI†). At the lowest temperature we observe that the average bonding is slightly higher for higher packing fractions, as in denser systems, every particle has more potential bonding partners available. At intermediate temperatures, instead, the peak in the average bonding shifts roughly in accordance with percolation line: for low *ϕ*-values the peak occurs at relatively low temperatures and then gradually shifts to higher temperatures as *ϕ* increases. At the highest temperature, the average bonding becomes smaller than two and is significantly higher for higher packing fractions. We interpret the described non-monotonous behavior in terms of effective particle functionality: far below percolation the percentage of branching points is very low with respect to the chain elements, while in a – arguably broad – temperature range around the percolation point it significantly increases, and it further goes down towards the monomer/dimer scenarios far above percolation.

Having ascertained that the average degree of bonding is very similar across the different systems, we wonder whether the visually observed different motives lead to different average bonding affinity for parallel or non-parallel. We calculate the fraction of parallel and non-parallel bonds and use it to devise an order parameter 1 − 2 × *P*_np_, where *P*_np_ is the fraction of non-parallel bonds. The parameter is −1 if all bonds are non-parallel and +1 if all bonds are parallel. In Fig. 7 (panels (a)–(d), second row) we report 1 − 2 × *P*_np_ for each system with *Δ* = 0.2 as a function of *T* for different *ϕ*-values. Again, we observe a non-monotonous behavior in temperature in each system at every density: while at low and intermediate temperatures, 1 − 2 × *P*_np_ is close to zero, at higher temperatures a maximum develops, signalling the onset of a specific bonding pattern. In particular, dma-as1 particles tend towards non-parallel bonding already at high temperatures, *i.e.*, in the fluid phase. In this system, the np-pattern becomes more pronounced for *T* = 0.10–0.14, such that 1 − 2 × *P*_np_ reaches values between −0.6 and −0.3. The reason for this highly non-parallel bonding is the appearance of very stable three particle loops bonded in a non-parallel fashion, before referred to as open boxes (see 3-np in panel (a) of Fig. 3). At low temperatures the bonding pattern becomes more mixed with a slight tendency towards parallel bonds (see chain elements such as 3-p-bs and 3-p-bs & np in panel (a) of Fig. 3). Similarly, the dmo-as1 system shows a mixed bonding scenario at low and intermediate temperatures, that is again associated to mixed patterns in chain elements. In contrast to dma-as1, dmo-as1 particles show a slight tendency towards parallel bonds on increasing *T*, which we associate again to the formation of finite clusters with a specific geometry – later discussed in more details. For dmo-s1 and dmo-s2 systems we observe similar trends: more mixed scenarios at low temperatures due to the mixed nature of the network elements and a peak of np- and p-bonds, respectively, at intermediate and high temperatures due to clustering. Some differences with respect to the previous systems can be appreciated, in particular in the low temperature regime where a tendency to maintain either the np- or p-patterns develops depending on the packing fraction. In particular in the dmo-s1 system, while we observe an ample non-parallel bonding at high and intermediate temperatures, with a maximum value of 1 − 2 × *P*_np_ between −0.4 and −0.2, at low temperatures bonding becomes mixed only for low packing fractions and remains non-parallel for high packing fractions. In contrast, dmo-s2 particles tend to maintain the tendency towards parallel bonding observed at intermediate temperatures – with maxima in the order parameter 1 − 2 × *P*_np_ between 0.3 and 0.4 – at low temperatures and low packing fractions, while they tend towards a mixed pattern on increasing packing at low temperatures. The described behaviour at low temperatures can be more clearly appreciated in the dependency of 1 − 2 × *P*_np_ on *ϕ* reported for the lowest investigated temperature *T* = 0.01 for all particle topologies with *Δ* = 0.2 in panel (e) of Fig. 7. Far below percolation, the mixed behavior is characteristic of dma-as1 and dmo-as1, while dmo-s1 and dmo-s2 maintain some preference towards either np- or p-bonding, respectively. It is worth stressing that, while the preference of dmo-s1 particles towards np-bonding develops on increasing *ϕ*, the preference of dmo-s2 particles towards p-bonding decreases with *ϕ*. For the other *Δ* values, *Δ* = 0.3 and *Δ* = 0.4, quantitative differences to *Δ* = 0.2 can be observed in 1 − 2 × *P*_np_ as function of temperature, but the general parallel/non-parallel bonding trends remain stable across all but one system, dmo-s1, where an additional peak indicates an abundance of parallel bonding for intermediate temperatures (see ESI† for details). In summary, below percolation we observe different degrees of mixed bonding patterns for all systems, while in the vicinity of the percolation some specific bonding patterns appear that we associate to the emergence of finite clusters with a specific geometry.

To quantitatively characterize the emerging finite clusters with their multi-particle bonding motives we calculate the average number of characteristic particle loops at every state point, spanning from clusters of size three up to size six. It is important to note by particle loops we refer to loops in the network graph. While, in the physical network all pores stem from particle loops, there are particle loops that do not form pores.

**Fig. 8:**
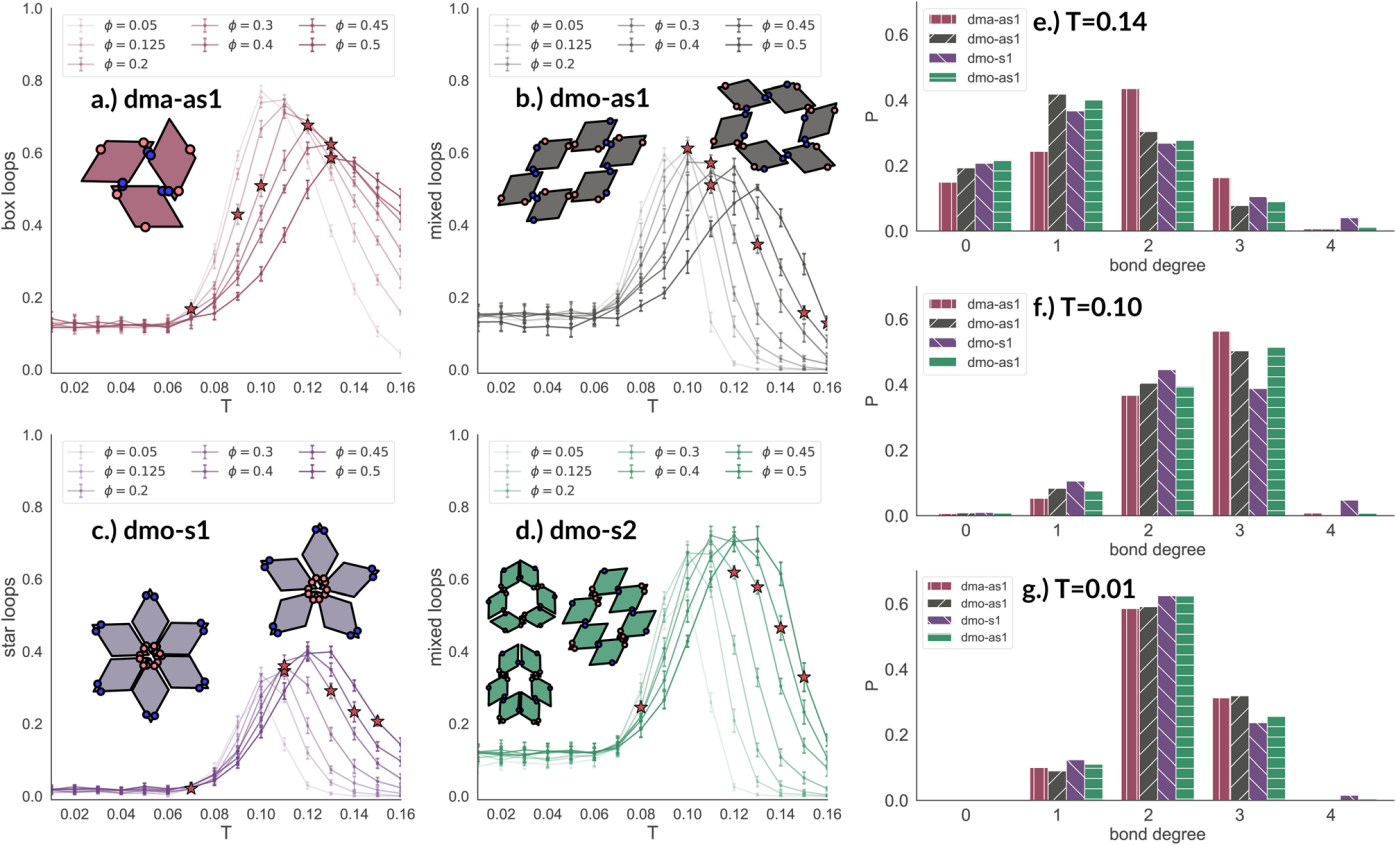
Fraction of particles bonded in prevalent loop types for all particle types at patch position *Δ* = 0.2 and packing fraction *ϕ* = 0.2 as function of temperature *T*. Panel (a): fraction of dma-as1 particles bonded in open box loops (labeled as 3-np in Fig. 3). Panel (b): fraction of dmo-as1 particles bonded in parallel and mixed (parallel and non-parallel) 6-particle loops. Panel (c): fraction of dmo-s1 particles bonded in non-parallel 5- and 6-particle loops. Panel (d): fraction of dmo-s2 particles bonded in parallel and mixed (parallel and non-parallel) 6-particle loops. Red stars highlight the state point at which percolation occurs. Bond degree histogram for all particle types with *Δ* = 0.2, *ϕ* = 0.2 and (e) *T* = 0.14, (f) *T* = 0.10 and (g) *T* = 0.01.

In Fig. 8 we report the percentage of particles bonded within the most characteristic clusters in each system with *Δ* = 0.2 as a function of *T* at different *ϕ*-values. In the dma-as1 system, the most characteristic particle loops are clusters of three particles with np-bonds only – already referred to as open boxes before (see their cartoon reported in panel (a) of Fig. 8). These loops are incredibly stable: they are abundant already above percolation (the higher the packing, the higher their occurrence), in the vicinity of the percolation 80% of particles are part of an open box cluster and about 20% of all particles are within boxes even well below percolation. It is worth noting that the peak is more pronounced at low packing fractions and it becomes less pronounced on increasing *ϕ* albeit still remaining significant at the highest investigated packing. In the dmo-as1 system, characteristic particle loops consist of six particles and can have either only p- or mixed bonds (see their cartoons reported in panel (b) of Fig. 8). Again, these clusters are present at all investigated state points, but they are more abundant in the vicinity of percolation where they reach up to about 60% at the lowest *ϕ* and about 50% at the highest *ϕ*. In the dmo-s1 system, characteristic particle loops consist of six or possibly five particles – so called star-clusters^59^ – and are characterized by np-bonds only (see their cartoons reported in panel (c) of Fig. 8). In contrast to the previous cases, the percentage of characteristic clusters is negligible below percolation and become abundant only in the vicinity of the percolation, where it reaches up to 40%, to again reduce to 20% at high temperatures. Interestingly, the height of the peak now increases with *ϕ*, meaning that stars are more abundant at higher densities. Finally, in the dmo-s2 system, p- or mixed-bonded particle loops (see their cartoons reported in panel (d) of Fig. 8) characterize the clustering region in the vicinity of percolation reaching up to 65–70%. Below the percolation their presence is reduced to 10%. Taking also the other *Δ*-values into consideration, we note that for dma-as1, dmo-as1 and dmo-s2 the looping behaviour stays qualitatively the same, with the box loops dominant in dma-as1 and p-bonded and mixed-loops dominant in dmo-as1/dmo-s2. In dmo-s1, on the other hand, for *Δ* = 0.3 and *Δ* = 0.4 p-bonded and mixed loops become prevalent (see the ESI† for loop plots for *Δ* = 0.3 and 0.4).

The distributions of the number of bonds (bond degree) for systems with *Δ* = 0.2 at the intermediate density *ϕ* = 0.2 – reported in panel (b) of Fig. 8 – add insights on the details. At *T* = 0.14, we observe that in each system particles have mostly one or two bonded neighbours, monomers are about 20% and are on average more abundant than particles with three bonds, four bonded neighbours are possible but rarely appear. It is worth noting that, the number of bonded neighbours distribution of the dma-as1 system differs on average with respect to the other ones: in particular, the relative higher percentage of particles with two bonds in the dma-as1 system with respect to the other systems is due to the formation of the boxes. On lowering the temperature to *T* = 0.10 – that is the temperature around which the maximum average of bonds per particle is typically observed, see Fig. 7 – monomers and singly bonded particles reduce quite substantially in all systems, and the large majority of particles forms two or three bonds. This feature hints at the formation of the finite clusters depicted in panel (a) of Fig. 8 (where two bonds per particle are needed) and at the possibility that these clusters connect, resulting in an extended (and possibly percolating) network. At the lowest temperature *T* = 0.01, particles have mostly two or three bonds, where the first ones are about 60% and the latter ones about 30%. It is worth noting that, in all systems, the percentage of monomers is going to zero on decreasing *T*, while a small amount of fully bonded particles is present at every temperature, with a relative higher abundance at high and intermediate temperatures: as discussed already in the small cluster analysis (see Fig. 3) for the very asymmetric patch topologies of *Δ* = 0.2 a fully bonded particle cannot be bonded to another fully bonded particle because of either steric constraints or mutually repulsive patches. While this scenario holds for *Δ* = 0.3, for the more symmetric *Δ* = 0.4, fully bonded clusters do occur for a limited number of intermediate packing fractions and densities (see ESI† for bond degree distributions for *Δ* = 0.3 and *Δ* = 0.4).

### Pore measures

Having discussed the properties of the networks and how they relate to the physical bonding motives, we now want to discuss how these affect the pore properties at different length scales. Pore properties such as pore size, pore circumference and pore asymmetry are the physical properties that are actually relevant for networks of organic molecules and colloids, as they define their quality and nature as filters (filter size, shape, and throughput, for instance), carbon-capture devices, surface modifiers and devices.

To analyse the pore properties we have developed an algorithm^55^ that splits the simulation box into volumetric elements – voxels – in our case squares of a certain length *l*_v_. We then check with the help of overlap algorithms how much of each voxels' area is occupied by particles. If that value is larger than a certain threshold *t*_occ_ we define the voxels as filled, otherwise they are empty. We then form a network with empty voxels as nodes and with edge connections established between empty voxel neighbours – including neighbours across the periodic boundaries. From the voxel network, we obtain the pores as lists of voxels by calculating all connected components with Networkx. We can then determine the size and circumference of the pores. We choose voxels' length *l*_v_ ≈ 0.25 as this is small enough to resolve the smallest pores, but large enough such that the algorithm still runs fast enough. The efficiency of the algorithm plays a large role here, as there is a huge number of data points to be evaluated. Note that *l*_v_ varies slightly for different packing fractions, with an estimated variation of ≈0.005 as the voxels always need to tile space. For the threshold *t*_occ_ we choose 0.25, but we checked that for the chosen *l*_v_, changes in *t*_occ_ do not significantly affect the results. For details on the algorithm and the choice of parameters see the ESI.†

**Fig. 9:**
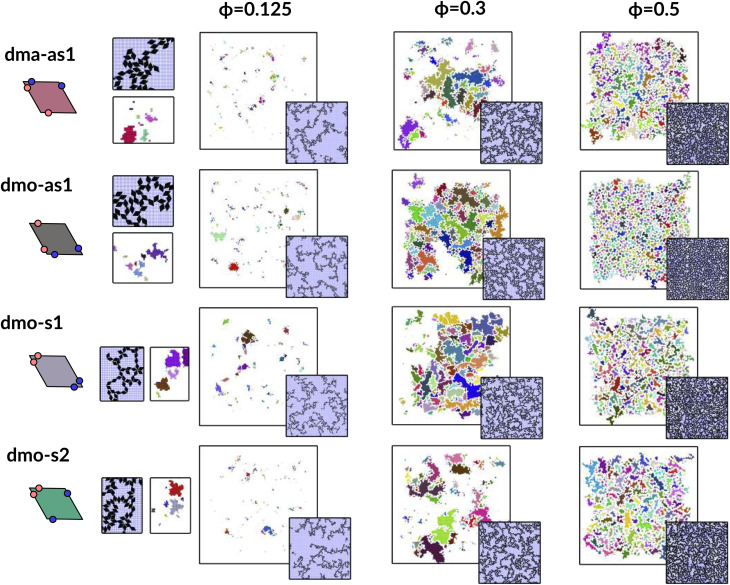
Snapshots of pores as identified by the voxel method. All snapshots show configurations with patch position *Δ* = 0.2 and at temperature *T* = 0.01, at different packing fractions *ϕ* = [0.125, 0.3, 0.5] (columns) for all particle types (rows); from top to bottom: dma-as1, dmo-as1, dmo-s1 and dmo-s2, as labelled. For better identification, separate pores are drawn in different colors, the void space – *i.e.* pores over the size of 2000 voxels are not shown, and pores that extend beyond periodic boundaries are stitched together. It is important to note that pores larger than 2000 still do enter the pore area and pore circumference analysis (see Fig. 10 here and Fig. S10 in the ESI†). Snapshot insets show the corresponding voxelated space with the particles visible. For every particle type, zoom-ins provide a more detailed view on exemplary pores at *ϕ* = 0.125.

**Fig. 10:**
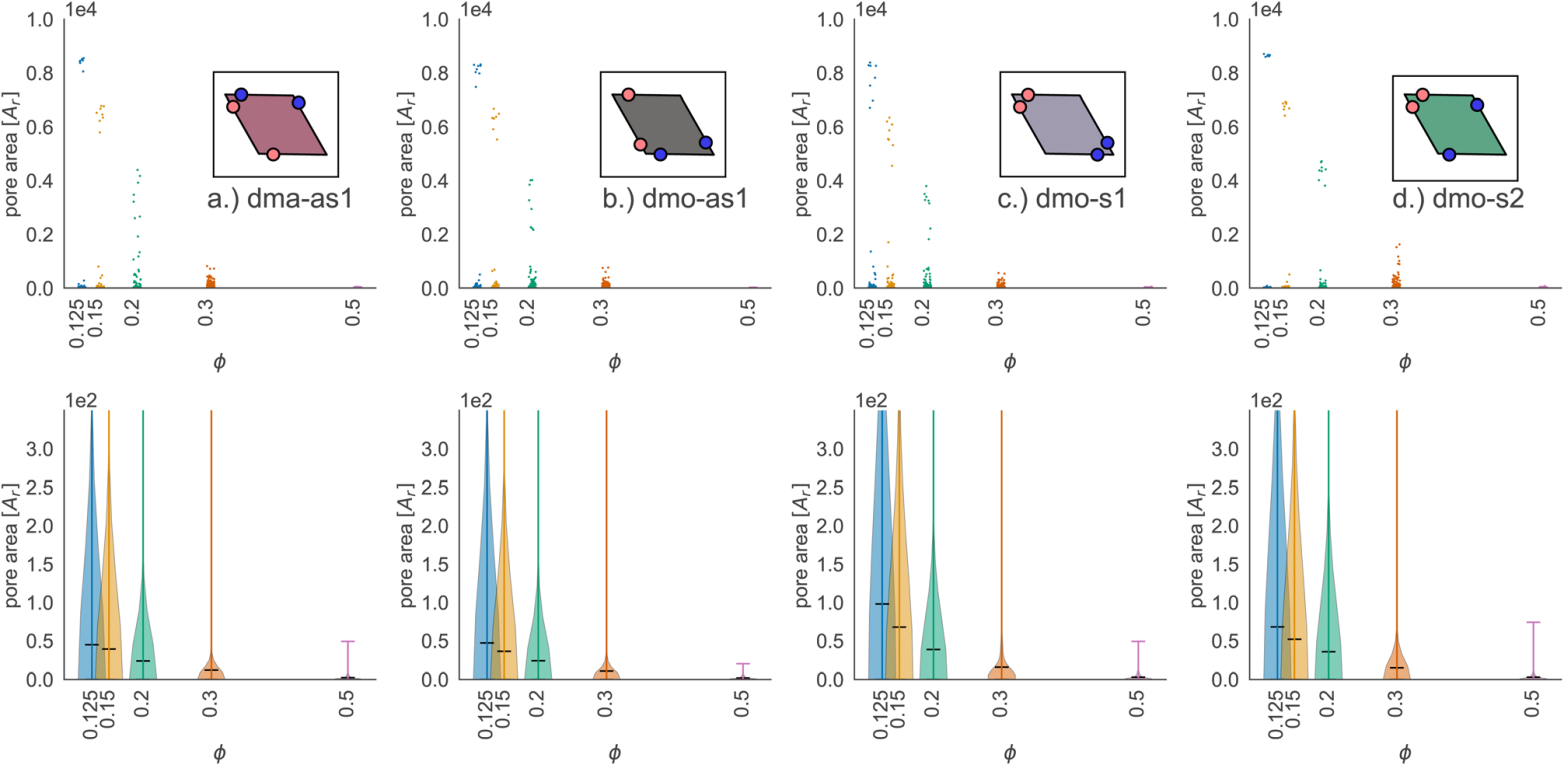
Pore area scatter plots (top row) and pore area kernel-density plots (bottom row) for particle types (a) dma-as1, (b) dmo-as1, (c) dmo-s1 and (d) dmo-s2 with *Δ* = 0.2 at packing fractions *ϕ* = [0.125, 0.15, 0.2, 0.3, 0.5]. For better visibility of the scatter, we added random noise to each scatter point in the *x*-direction.

In Fig. 9 we highlight the detected pores for particles with *Δ* = 0.2 at the lowest investigated temperature *T* = 0.01 at three different densities, *ϕ* = 0.125, *ϕ* = 0.3 and *ϕ* = 0.5; all chosen state points are percolating. Note that different pores are associated to different colors and that pores larger than 2000 are left white. At *ϕ* = 0.125, we observe a range of small and mid sized pores, and a large void space. This split distribution of sizes can be appreciated in the pore size scatter plots of Fig. 10 (blue scatter), where most pore sizes lie below 200, representing the small (<20 in units of the particle area) and mid-size (20–200) pores, while some lie at 8000, representing the void. As the density increases – *ϕ* = 0.15 (yellow scatter) and *ϕ* = 0.20 (green scatter), the size difference between void and pores decreases until it almost closes at *ϕ* = 0.3 (orange scatter), which can be also visually appreciated in the corresponding panels of Fig. 9, where due to the higher density, the void space has been split up into multiple mid-size pores, and only one sightly larger white area representing the void is left. For the highest density *ϕ* = 0.5, the pores are split further into smaller regions by the now denser particle network (see the corresponding panels in Fig. 9 as well as the pink scatter of Fig. 10).

Taking a closer look at the mid-size pores (size 20–200), we can extrapolate some differences between the different systems by performing a kernel density estimation (KDE), which is a parameter-less method of estimating an unknown probability distribution from the data. The results are shown as violin plots in panel (b) of Fig. 10 – with the means of the estimated densities shown as black ticks. While for *ϕ* ≥ 0.2 kernel densities are similar across the different systems, for *ϕ* < 0.2 we see, that the kernel densities of dma-as1 and dmo-as1 have significantly shorter tails than those of dmo-s1 and dmo-s2. On the one hand side, this can be seen from comparing the tail thickness at a given pore size: in particular, at large pore size values, the tails of dmo-s1 and dmo-s2 still show a visible thickness where the tails of dma-as1 and dmo-as1 have already thinned. On the other hand, the same feature can be observed from mean pore size estimated from KDE, that is consistently larger for dmo-s1 and dmo-s2 for *ϕ* < 0.2. At *ϕ* = 0.125 the estimated mean for dmo-s1 is around 100, for dmo-s2 it is around 65, while for dma-as1 and dmo-as1 it is around 30.

**Fig. 11:**
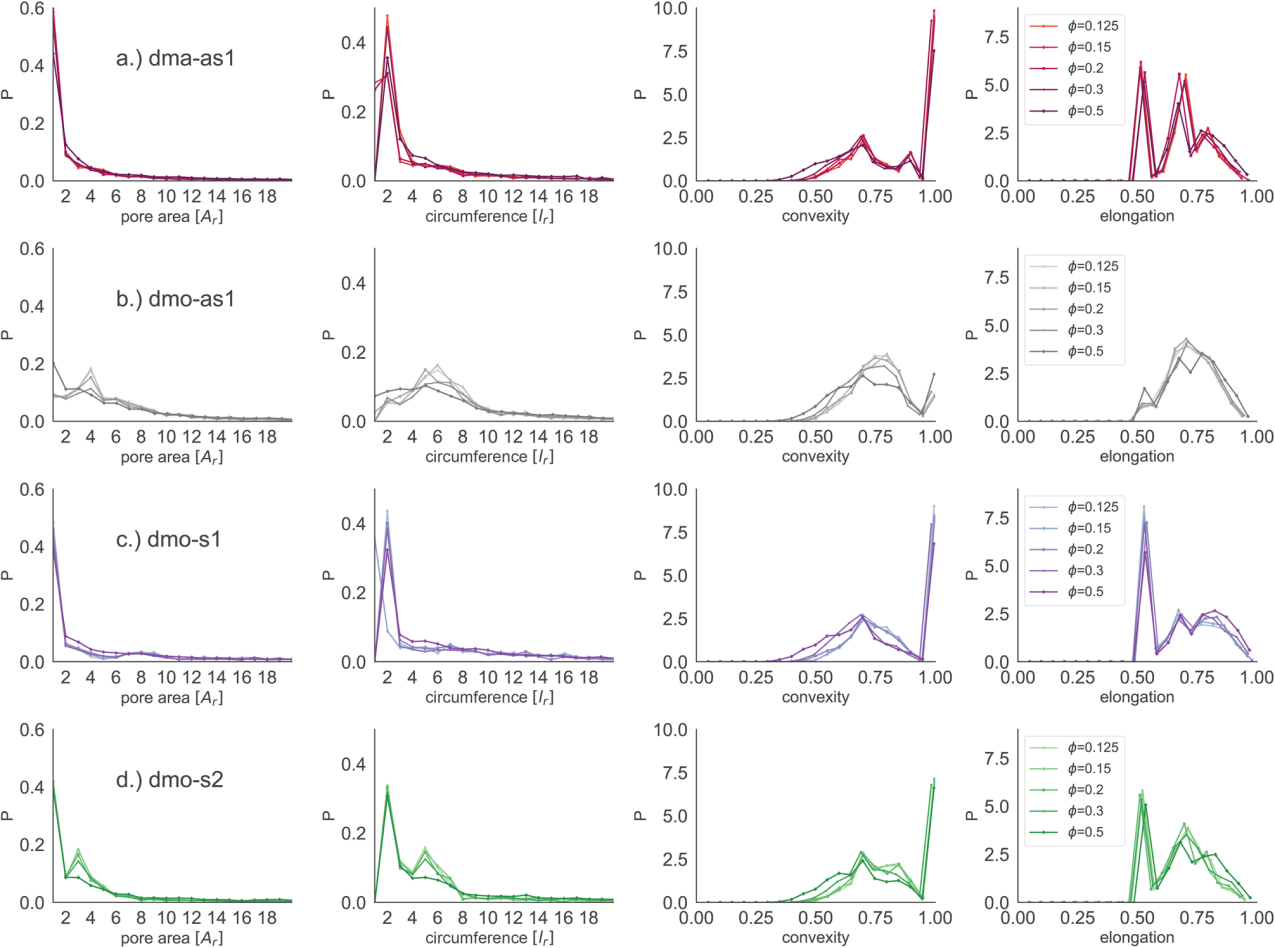
Row-wise from left to right: pore area, pore circumference, pore convexity (measured by comparing pore area with the pore's convex hull) and pore elongation (measured by the percent of explained variance in the first principal component *p*_PCA1_) for particle types (a) dma-as1, (b) dmo-as1, (c) dmo-s1 and (d) dmo-s2 with *Δ* = 0.2 at different packing fractions *ϕ* = [0.125, 0.15, 0.2, 0.3, 0.5] and temperature *T* = 0.01. Units for pore area and pore circumference are given in terms of particle area *A*_r_ and particle side length *l*_r_, respectively.

Focusing on small pores (size 1–20), we report the pore area and the pore circumference in panels (a) and (b) of Fig. 11: all systems have their own set of typical pore areas/circumferences that are represented as peaks and valleys with specific positions, heights and widths in the pore area/circumference distributions (see the ESI† for details on how we measure the pore circumferences). In particular, dma-as1 has one dominant peak at pore area one and circumference two, hinting at the abundance of small pores. The peak in one for the pore area is also present for dmo-as1, but it is much less dominant and another dominant peak appears at pore area four, that is strong at low densities *ϕ* = 0.125 and *ϕ* = 0.15, but becomes weaker as the density increases. For low densities the circumference has two peaks, one at two and one at six while for larger densities, the two distinct sharp peaks become one broad peak. For dmo-s1 only the peak at one emerges for the pore area, again paired with a circumference peak at two. For dmo-s2, there are two dominant pore areas, one and three, that come together with a circumference of two and a circumference of six. For pore sizes up to 2000, we also studied the asymmetry of the pores and the results are summarized in panels (c) and (d) of Fig. 11. In panel (c) we compare the pore area to the respective convex hull of the pores, a quantity that we label as convexity. This measures gives in fact a coarse idea of how “fingered” (*i.e.*, how non-convex) the pores are: with a value of one, the pores are of a convex shape, and the closer the value is to zero, the less convex, or more “fingered” the pores become. For this analysis we calculated the convex hull, with the qhull package.^60,61^ In panel (d) we perform a principal component analysis of points sampled inside the pores with the scikit-learn package,^62^ and plot the extent to which the first component explains the variance in this scatter data, a quantity we label elongation: if this value is closer to one, the first component explains all of the variance and the pore is rather elongated, while the closer the value is to 0.5 the more disk-shaped the pore is. For these two asymmetry measures we observe that each systems shows a typical peak-and-valley pattern that persists over all densities, where the peak positions are similar for all systems, but the peak heights and widths are particular for each system. For the convexity measure, there are three peak positions that recur, one at ≈0.67, a smaller peak at ≈0.85 and a high peak at ≈1.0. For the elongation measure, we also see three peaks, one at ≈0.5 one at ≈0.7 and one at ≈0.8. For dma-as1, all three peaks are clearly distinguishable for the convexity as well as the elongation. For dmo-as1, the first and second peak are combined to one broad peak for the convexity and we observe all three peaks in the elongation for high densities, while for low densities the second and the third peak are combined. For dmo-s1, for the convexity measure only the 0.68 and the peak at 1 are clearly visible, while all three peaks in the elongation are distinguishable, albeit the second and third peak are not as separable as the first. For dmo-s2, the first and second peak in convexity are visible but not clearly separable, while third peak is clearly distinguished, in the elongation measure the first peak is clearly separated, while second and third are combined. To understand which pore shapes lead to these particular asymmetries, we conducted an in-depth analysis where we investigated the convexity and the elongation for different pore sizes. We found that the typical peaks are due to pore sizes smaller than 20 in units of voxel number (see ESI†).

## 4 Conclusions

In this work we investigated the effects of bond and shape anisotropy on the network properties of two-dimensional self-assembling systems. The selected set of coarse-grained systems represents elongated, functionalized molecules and their assembly in two dimensions – investigated by means of extensive Monte Carlo simulations in the canonical ensemble – reproduces the assembly of the molecules on a non-interacting substrate. In particular, we consider rhombic platelets arbitrarily decorated with four bonding sites – or patches – along their edges, with two of these patches being mutually repulsive and two of them mutually attractive, and we consider a subset of four different patch topologies (dma-as1, dmo-as1, dmo-s1 and dmo-s2), each characterized by a parameter (*Δ*) that controls the asymmetry of the given topology. In molecules, different patch types can be realised by using different functional groups or by exploiting different deprotonation levels according to the pH, while different patch arrangements (each fixed by a topology and a value of the asymmetry parameter) correspond to different molecules.

On the basis of statistically robust data, collected by means of massive many body simulations (large systems, parallel runs), we were able to characterize the emergence of extensive particle networks. We described in detail the network properties such as the connectivity and the percolation locus, the clustering behaviour associated to the typical local bonding motives, as well as the geometric properties of the networks such as the pore size and shape. We also proposed a straightforward interpretation of these emerging features in terms of steric incompatibilities and energetically unfavourable patterns by putting forward a detailed small cluster analysis.

In summary, we observed that the emergence of a percolating cluster of particles occurs in the same range of densities and temperatures for the whole set of investigated systems, despite of the bond motives differing quite substantially from one topology to another. The similarity of the percolation behavior is also confirmed by the connectivity analysis: this highlights that networks of different systems have the same branching behaviour. Another common feature observed is the tendency of particles to form more bonds in the vicinity of the percolation; in particular, the number of bonds per particle has a non-monotonic behavior in temperature in the whole range of investigated densities: below percolation each particle forms on average two bonds only, while in the vicinity of the percolation the number of bonds per particle grows and can be as high as three, while on further increasing the temperature the number of bonds becomes low again and reaches values between two and zero – depending on the density. It is worth noting that, despite the fact that in some systems fully bonded particles can exist, fully bonded assemblies cannot emerge – not even at the highest reached packing fraction (which is *ϕ* = 0.5). We rationalized this by showing that a fully bonded particle cannot have fully bonded neighbours because of steric incompatibilities or repulsive interaction patterns. We characterized this non-monotonic behavior by isolating the different clustering motives of the different systems and showing that their relative abundance has a non-monotonic behavior. Finally, we characterized the porosity of the observed assemblies. The investigated features – pore size distributions, pore area, circumference, convexity and elongation – highlight system-specific patterns.

In conclusion, the networking properties of these systems are a robust feature that is independent of the specific balance between anisotropy in bonding and anisotropy in shape, while the bonding and looping motives – that lead to the percolating networks – are strongly dependent on the topology of the functionalized sites. This suggests that the porosity of molecular disordered assemblies on an inert substrate is tightly related to the choice of the molecules, while the formation of extended and branched two dimensional clusters might occur in the same range of thermodynamic parameters for a broad variety of different molecules.

## Conflicts of interest

There are no conflicts to declare.

## Supplementary Material

NA-006-D3NA00621B-s001

NA-006-D3NA00621B-s002
