# Supplementary material for: Anisotropic functionalized platelets: percolation, porosity and network properties
Source: Nanoscale Adv. 2023 Dec 11;6(2):443–57. doi: 10.1039/d3na00621b (PMC10790971; doi:10.1039/d3na00621b)
Supplement: NA-006-D3NA00621B-s001 [file NA-006-D3NA00621B-s001.pdf]

Electronic Supplementary Material (ESI) for Nanoscale Advances.
This journal is © The Royal Society of Chemistry 2023

# Supporting Information for Anisotropic functionalized platelets: percolation, porosity and network properties

Carina Karner* and Emanuela Bianchi†
(Dated: November 14, 2023)

## I. METHOD DETAILS

### A. Network properties

For calculating certain network properties only the bonding information of the particle network matters, while physical properties of the bonded particles – the particle positions and orientations and bonding angles — can be ignored. Thus, the aggregate can be treated as a non-physical network, where each particle becomes a network node and each bond between two particles becomes an edge connecting two nodes. With this network representation we calculate the bond degree distribution, the percolation line and the percentage of particles within network loops of a certain size. We obtained these network properties with our own software – partially published here [1] – that relies on Pythons Networkx module [2].

#### 1. *Network degree distribution*

We used Networkx' degree() method to return the list of node degrees and subsequently calculated the degree distribution, the average degree and its standard deviation. The average bond degrees of all particle types are featured in Fig. 7 and the bond degree distribution in Fig. 8 within the main text.

#### 2. *Percolation line*

A percolating cluster is defined as cluster of bonded particles that extends beyond the periodic simulation box to infinity in at least one direction. To test for percolation in practice, we calculate the largest particle cluster over all nine periodic images of the simulation box and define the cluster as percolating if more than 0.33 of all particles are in this largest cluster. We chose this threshold of 0.33, because in that case the percolating cluster spans across the periodic images in at least one direction. To calculate the largest cluster of all periodic images, we first enhance the network to include the particles and bonds – i.e., the nodes and edges – of all nine periodic images. Note that, to build this large network efficiently, the only additional information needed is the connectivity between two periodic images, as for non boundary particles we can just copy the edge connectivity from the original simulation box. We calculate the largest cluster of this network of periodic images by calling Networkx' method *connected_components()*, which returns all connected components, i.e., all clusters, as lists of nodes; the largest of these lists, is then the size of the largest cluster. If the relative size of this largest cluster of all periodic images, $\frac{R}{N*9}$ – where $R$ is the size of the largest cluster, and $N$ is number of particles within the original simulation box – is larger than than 0.33 the cluster is defined as percolating. We define the percolation locus for each density as the temperature where configurations in at least half of the parallel runs contain a percolating cluster; The percolation line is the line that connects the percolation points across all densities. See Fig. 6 in the main text for a depiction of the percolation lines for all studied systems.

#### 3. *Network Loops*

. We detect particle loops of sizes three to six by exploiting Networkx' *all_simple_paths()* method: with *all_simple_paths()*, all paths between two nodes $i$ and $j$ of given a length $l$, that do not contain repeated nodes,

*Electronic address: carina.karner@tuwien.ac.at
†Electronic address: emanuela.bianchi@tuwien.ac.at

can be obtained. We therefore deduce, that all simple paths $l > 1$ between two neighbouring nodes must be loops. Hence, all loops of a specific size $l$ can be obtained by calculating all simple paths between all neighbouring nodes. It is important to note that with this algorithm all loops of size $l$ are counted $l$ times, therefore the obtained loop counts have to be normalized. To distinguish between loops with only parallel (p), non parallel (np) or mixed loops, with a mixture of p- and np-bonds, we add the bonding type (p, np) as an edge property to our network and filter the network for the desired bond-type (p, np, mixed) before evaluating all simple paths. In Fig. 8 within the main text we show the fraction of particles within the most dominant loops as function of temperatures for all particle types at the patch position $\Delta = 0.2$ and a selection of packing fractions $\phi$=[0.05,0.125,0.2,0.3,0.4,0.45,0.5]. Results for $\Delta = 0.3$ and $\Delta = 0.4$ are depicted within the Supporting Information Fig. 3 and Fig. 4.

## B. Pore properties

We identify and analyse the porosity of networks with specially devised methods. Some of the used algorithms can be found in our github repository [3].

### 1. *Voxel method*

The first step in pore analyses must be the identification of the pore space - i.e., the area of the simulation box occupied by pores, where a pore is defined as an isolated subspace surrounded by particles. For this purpose, we developed a volumetric-element (voxel) based method, where the simulation box is split into small squares (voxels) of a side length $l_v$, where $l_v$ is significantly smaller than the side length of a rhombus particle $l_r$. We chose $l_v \approx 0.25$, as this size enables us detect the smallest pores, while still allowing us to evaluate the pores for all state points efficiently. Here it is important to note that $l_v$ varies slightly for different packing fractions, with an estimated variation of $\approx 0.005$ as the voxels always need to tile space. The voxels are arranged in a square lattice, with eight neighbours per voxel, where four neighbours share an edge and four share a corner. Periodic boundary conditions apply and next neighbours of voxels at the simulation box edge are given through minimum image convention. For each voxel $vx_i$, we estimate the area occupied by rhombus particles. Here, cell-lists ensure efficiency in calculating potentially overlapping rhombi $rh_j$, where rhombus-center-to-voxel-center distances are small enough for the shapes to possibly overlap. For each of these overlap candidates, we estimate the area of overlap $a_{ij}$ by randomly drawing $m = 100$ points from the voxel $vx_i$ and checking if they overlap with a given rhombi $rh_j$. This overlap-detection procedure is repeated for all neighbouring rhombi of voxel $vx_i$, and the in total occupied area for $vx_i$ is obtained by summing up $a_i = \sum_{j=0}^{Nb_i} a_{ij}$ where $Nb_i$ is the number of overlap candidates for voxel $vx_i$. The given voxel $vx_i$ is set to a filled state if the occupied area $ai$ exceeds a threshold $t_{\rm occ} = 0.25$ and remains in an empty state otherwise.

Note, that we evaluated the pore space also for $m = 500$ and $m = 1000$ sample points instead of $m = 100$, and also for different overlap thresholds $t_{\rm occ} = [0, 0.1, 0.25, 0.3]$ and found no significant difference.

The pore space can be finally calculated by forming a network of empty voxels, where every empty voxel is a node and every connection with another empty neighbouring voxcel is an edge; from this empty voxel network we obtain the pores as lists of voxels by invoking Networkx's *connected_components()* method. The snapshots in Fig. 9 in the main text highlight the pores in different colors, while the insets show voxel space with all voxels framed in blue.

### 2. *Pore area*

From the voxel method, we obtain the pores as lists of voxels and the pore area (pore size) distribution can be obtained by calculating the length of these list and multiplying it with the voxel area $a_v = l_v^2 \approx 0.625$. For Fig. 10 and 11 in the main text, we further divide each pore area $A_p$ by the rhombus area $A_r = 0.866025$, as this gives us the pore area in units of the particle area.

### 3. *Pore circumference*

The circumference $\mathrm{Cf}_m$ of a pore $p_m$ built from $K$ voxels $vx_k$ can be obtained by summing up all exposed edges per voxel,

$$\mathrm{Cf}_m = \frac{l_v}{l_r} \sum_{k=0}^{K} 4 - N_{\mathrm{edge},k} \tag{1}$$

where $N_{\text{edge},k}$ is the number of edge-to-edge voxel neighbours of voxel $vx_k$ within that pore, $l_v$ is the side length of a voxel and $l_r$ is the particle side length. The pore circumference of all particle types at patch position $\Delta = 0.2$ and temperature $T = 0.01$ can be appreciated in Fig. 10, Panels (a)-(d).

#### *4. Pore asymmetry measures*

To estimate the asymmetry of the pores we use two measures, with one measuring the convexity and the other one the elongation of the pores.

In contrast to the pore area and the circumference, that were evaluated algebraically, for the pore asymmetry measures the actual pore shape matters. Therefore, as pre-evaluation step, pores that extend beyond the periodic boundaries, must be stitched together. We perform the stitching of a boundary pore $p_m$ by randomly selecting a voxel $n$ within the pore, and then chose the periodic images of all other voxels to be closest to that voxel.

As this method breaks when the extent of a pore grows beyond half of the simulation box, we restrict ourselves to pores smaller than 2000 voxels.

To evaluate the convexity of a pore $p_m$, we compare the actual pore area $A_{p,m}$, to the area of its convex hull $A_{\text{convex},m}$. We calculate the convex hull $A_{\text{convex},m}$ of a pore $p_m$ by forming a point cloud of center and corner positions of all voxels contained in that pore and then use this point could as input calculation of the convex hull with the qhull package [4, 5]. We obtain the convexity measure, $A_{p,m}/A_{\text{convex},m}$ as a number between 0 and 1, where values close to 0 indicates highly non-convex pores, while 1 represents convex pores. Generally, pores with a high convexity tend towards elliptic or circular shapes, while pores with a high convexity exhibit many dents and fingers.

To evaluate the pore elongation we perform a principal component analysis (PCA) with the aid of Pythons Machine Learning package scikit-learn [6].The input data for each pore $p_m$ is the point cloud of all voxel positions and corners contained in $p_m$. We use the percent of explained variance of the first principal component, $p_{PCA1}$, as measure for the pore elongation: if $p_{PCA1}$ is close to 1, most of point-cloud's variance can be explained by the first – the dominant – component, *i.e* the clusters are very elongated, while if $p_{PCA1}$ is closer to 0.5, the point-cloud's shape is more spherical.

In Fig. 11 in the main text, the pore convexity (Panels (a)-(d), third column) and the pore elongation (Panels (a)-(d), fourth column) are plotted for all particle types at patch position $\Delta = 0.2$ and for different packing fractions $\phi = [0.125, 0.15, 0.2, 0.3, 0.4, 0.5]$ at temperature $T = 0.01$. Additionally we show the convexity and elongation binned per size ranges here in the Supporting Information in Fig. 11 and Fig. 12.

## II. ADDITIONAL DATA

### A. Average bonding affinity for $\Delta = 0.3$ and $\Delta = 0.4$

The order parameter $1-2P_{np}$, with $P_{np}$ as the fraction of non-parallel bonds, indicates the average bonding affinity of the system, where a value of -1 represents system with only non-parallel bonds and 1 designates a completely parallel assembly. While the main text discusses the bonding affinity of particle topologies with $\Delta = 0.2$ (see main text, Fig. 7, Panels (a)-(d), second row), here in the Supporting Information we show the results for $\Delta = 0.3$ in Fig. 1 and for $\Delta = 0.4$ in Fig. 2. For dma-as1, dmo-as1, and dmo-s2, we observe similar trends as in $\Delta = 0.3$ and $\Delta = 0.4$ as we do for $\Delta = 0.2$: For dma-as1 (Panel (a) of Figs. 1 and 2), we observe a dominance in non-parallel bonding already for high temperatures, that becomes even more prevalent in the broad intermediate temperature range around the percolation point. Well below the percolation point, non-parallel bonding diminishes and we observe more mixed bonding. In dmo-as1 (Panel (b) of Figs. 1 and 2), we generally observe more mixed bonding througout all temperatures, with only a slight predominance of parallel bonds at higher temperatures. For dmo-s2 (Panel (d) of Figs. 1 and 2), we observe a slight tendency towards parallel bonding for high temperatures. For intermediate temperatures, this parallel tendency becomes stronger for $\Delta = 0.3$, while for $\Delta = 0.4$ it remains minor, and for low temperatures the bonding affinity becomes more mixed again for both $\Delta = 0.3$ and $\Delta = 0.4$.

For dmo-s1, on the other hand we observe a qualitative change in the bonding affinity for $\Delta = 0.3$ (Panel (c), of Fig. 1), and $\Delta = 0.4$ (Panel (c), of Fig. 2), compared to $\Delta = 0.2$. In the main text (see Fig. 7, Panel (c), scond row), we had observed for $\Delta = 0.2$ more mixed bonding for high temperatures that transforms to more non-parallel bonding for intermediate temperatures, and then back again to more mixed bonding at low temperatures, where the affinity towards non-parallel bonding is stronger for higher densities, where it peaks at -0.5 for intermediate temperatures. For $\Delta = 0.3$ the tendency towards non-parallel bonding becomes significantly weaker, especially for the high densities, with a – now diminished peak – peak at $-0.2$ for high densities. For lower densities the propensity to bind non-parallel is even even further diminished and at the highest density only a slight tendency towards non-parallel

bonding is visible. For $\Delta = 0.4$, the tendency towards non-parallel bonding is only visible for higher temperatures, at intermediate temperatures, this tendency reverses, and parallel bonding becomes more prevalent instead. While this additional positive peak can we observed for all densities, it is strongest for low packing fractions, where it peaks at 0.3. At low temperatures, we again spot more mixed bonding across all densities.

### B. Particle Loops for $\Delta = 0.3$ and $\Delta = 04$

In the main text we analyse particle loops, i.e loops within the network graph, for $\Delta = 0.2$ and find that in the vicinity of the percolation points, a majority of particles are bound within loops of size three to six (see Fig. 8, Panels (a)-(d) in the main text). Here, in the Supporting Information we report the occurrence of the most dominant loops as function of temperature for $\Delta = 0.3$ (Fig 3, Panels (a)-(d)) and $\Delta = 0.4$ (Fig. 4, Panels (a)-(d)). Generally we find that for the dma-as1, the dmo-as1 and dmo-s2 system, the percentage and type of the dominant loops is qualitatively similar to $\Delta = 0.2$. More specifically, in the dmo-as1 system (Figs. 3 and 4, Panel (a)) non-parallel (np) particle loops of size three – boxes – make up the majority of present loops for all $\Delta$-values and packing fractions. Their occurrence at high temperatures is density dependent, and for high densities they already occur to large amount at the highest temperatures. For intermediate temperatures near the percolation points boxes occur at 60-80% for all densities and even far below the percolation line, about 20% of particles remain bonded in boxes across all packing fractions. For both dmo-as1 (Figs. 3 and 4, Panel (b)) and dmo-s2 (Figs. 3 and 4, Panel (d)), we observe either parallel (p) or mixed (p- and np-bonds) six particle loops as the dominant loop motives; at high temperatures we detect that about 20% of particles are bonded these motives for high densities, with the percentage decreasing progressively as the density becomes lower; for intermediate temperatures, be observe up to 80% of p-bonded and mixed loops for all densities and for low temperatures this amount drops to 20% for all packings. In contrast, in dmo-s1, $\Delta = 0.3$ (Fig. 3, Panel (c)) and $\Delta = 0.4$-systems (Fig. 4, Panel (c)) differ qualitatively from the $\Delta = 0.2$ case. While for $\Delta = 0.2$, the dominant loop motives are 5-np and 6-np loops – so called 5-stars and 6-stars – for $\Delta = 0.3$ mixed (with p- and np-bonds) six particle loops become more prevalent than stars for all temperatures and densities, and for $\Delta = 0.4$ it is p-bonded as well as mixed loops that overshadow the occurrence of stars. At high temperatures, mixed and p-bonded/mixed loops occur at a significant percentage for higher densities for $\Delta = 0.3$ and 0.4 respectively, but remain insignificant for lower densities; at intermediate temperatures 60-80% percent of particles reside within mixed and p-boned/mixed loops across all densities, while for low temperatures their amount drops to 10-20%. To illustrate these qualitative changes further, we show the percentages of box loops, star loops, parallel loops and mixed loops for dmo-s1 $\Delta = 0.2$ (Fig. 5), $\Delta = 0.3$ (Fig. 6) and $\Delta = 0.4$ (Fig. 7).

### C. Bond degree distribution for $\Delta = 0.3$ and $\Delta = 0.4$

In the main text, we report the bond degree distribution of all patch topologies for the patch position $\Delta = 0.2$ and the packing fraction $\phi = 0.2$ as well as three temperatures $T = 0.14$, 0.10 and 0.01 Here, in the Supporting Information we additionally discuss the bond degree distribution for $\Delta = 0.3$ in Fig. 8 and of $\Delta = 0.4$ in Fig. 9. We find that the bond degree distribution for $\Delta = 0.3$ qualitatively exhibits same behaviour as $\Delta = 0.2$: for high temperatures at $T = 0.14$ (Fig. 8, Panel (a)), we observe all bonding scenarios from zero to fully bonded (four), with the majority of particles bonded to only one neighbour particle. While dmo-as1, dmo-s1 and dmo-s2 show a similar bonding distribution, in dma-as1 we observe a higher percentage of two-bonds, which indicates the presence of the three particle box clusters already at high temperatures. At intermediate temperatures, at $T = 0.10$ (Fig. 8, Panel (b)), we observe that the majority of particles has two or three neighbours, which – as with $\Delta = 0.2$ – hints at the presence of connected clusters of loops described in Fig. 3. For the lowest studied temperatures, $T = 0.01$ (Fig. 8, Panel (c)), we find that two-particle bonds become the prevalent bonding type, which indicates the increased chaining of the network. In $\Delta = 0.4$, for $T = 0.14$ and $T = 0.01$ (Fig. 8, Panel (a) and (d)), the bond degree distribution remains qualitatively similar to $\Delta = 0.2$ and $\Delta = 0.3$. On the other hand for intermediate temperatures $T = 0.10$ (Fig. 8, Panel (b), while the majority of particles of all patch topologies still has two- or three neighbours, we spot a significantly higher amount of four-bonded particles for dmo-s1 and dma-as1. At visual inspection of simulation snapshots we find that some of these four-bonded particles reside in fully-bonded finite clusters. We conclude that for the more symmetric $\Delta = 0.4$ patch positions, the bonding strain due to the patch asymmetry is already reduced to such that fully bonded finite clusters become more favorable and do co-occur with two- and three-bonded finite clusters.

### D. Pore measures

#### 1. Pore circumference

Besides the pore area, which is discussed in the main text, we also evaluated the pore circumference in units of the rhombi side length $l_r$ according the formula in Eq. 1 at the lowest temperature $T = 0.01$ for the patch position $\Delta = 0.2$ and for different packing fractions $\phi = [0.125, 0.15, 0.2, 0.3, 0.5]$. Analogously to the pore area, the scatter plot of pore circumferences of all patch topologies at the lowest packing fraction of $\phi = 0.125$ (Fig. 10, Panels (a)-(d) top row, blue scatter) shows a split between pore circumferences smaller than 200 – representing the small and mid-size pores and pore circumferences of $> 4000$, representing the void space. As the density increases, the difference in circumference between void space and pore space becomes smaller, as the large void space is cut into smaller pieces by bonded particles sharing a smaller simulation box, and at $\phi = 0.3$ (Fig. 10, Panels (a)-(d) first row, red scatter), no significant difference is visible anymore between the circumference of void and pores. The kernel density estimation (KDE) – a parameter free estimation of the probability density – of the pore circumference (see Fig. 10, Panels (a)-(d), second row) shows that for low packing fractions of $\phi = 0.125$ and $\phi = 0.15$ the pore circumferences of dmo-s1 with an KDE-estimated length of around $\approx 50$ and – to a lesser degree – dmo-s2 with a length of $\approx 40$ are longer than the circumferences of dma-as1 and dmo-as1 with lengths of $\approx 30$.

#### 2. Dependency of asymmetry measures on pore size

In the main text we evaluated the convexity and the elongation of pores with pore areas smaller than 1000 (see Fig. 11, Panels (a)-(d), third and fourth column in the main text) voxels and found that, for both convexity and elongation, every particle type has its own typical peak patterns, discern-able by the relative height and sharpness of the peaks. Here, we investigate in detail which pore sizes are responsible for these unique peak patterns by evaluating the convexity and the pore elongation for pores of different size ranges. The results are shown in Fig. 11, Panels (a)-(d) for the convexity measure and in Fig. 12, Panels (a)-(d) for the elongation measure. It is important to note that, for this analysis the pore area is given as the number of voxels within a pore, as this enables us to better trace the asymmetry measures back to the pore shapes. Generally we observe that it is mainly the small pores, that are smaller than 20 voxels, that are responsible for the unique peak patterns in both measures. More specifically in the convexity measure (see Fig. 11, Panels (a)-(d), the peaks very close to one, indicating convex pores, are due to pores of sizes $< 10$, the peaks in the vicinity of 0.68 are due to pores between sizes 5 and 20, while the peaks near 0.85 appear for pores of size $10 - 20$. While these three peaks do occur in all patch topologies, the relative height of these three peaks differs and we argue that is this difference that largely defines the unique peak patterns for each system. For pores beyond the size of 20, the peaks at 0.68 and 0.85 broaden and become one wider peak, while the peak close to one – signifying rather convex pores – disappears. Finally, we observe that at the highest density (Fig 11, Panels (a)-(d), last column), the pore convexity is significantly lower for larger pores $200 - 1000$, representing a trend that can be spotted for all patch topologies.

Taking a closer look at the elongation at different size ranges (see Fig 12, Panels (a)-(d)), an analogous picture forms: for pores of sizes $< 5$, the elongation measures peaks at 0.5 – indicating rather symmetrical pore shapes and for pores between 5 and 10, this 0.5-peak vanishes, and instead two peaks – a larger one at 0.65 and a smaller one at 0.75 appear. While all patch topologies and densities show these exact distinct peaks for pores of size $0 - 5$ and size $5 - 10$ – albeit with different heights — for pore sizes between between 10 and 20 more distinguished peaks appear between 0.65 and 0.9, but in these cases, the exact number of the peaks as well as their position and width differs for different patch-topologies and densities. Here it is interesting to note, that despite these peak pattern differences for different densities in each system for pore sizes $10 - 20$, when evaluated over all pores sizes, a unique peak pattern is retained for each systems across all densities. Moving to pores $> 20$, we find a severe broadening of the distributions for all systems, to extent where no distinct peak can be identified. We interpret the results such that, while pores with sizes $< 20$ do have distinct shapes with unique peak patterns per particle type and density, larger pores do occur in all elongations.

[1] C. Karner, *GelAnalyser*, https://github.com/OljaOctagon/GelAnalyser (2023-2030).
[2] A. A. Hagberg, D. A. Schult, and P. J. Swart, in *Proceedings of the 7th Python in Science Conference*, edited by G. Varoquaux, T. Vaught, and J. Millman (Pasadena, CA USA, 2008), pp. 11 – 15.
[3] C. Karner, *PoreAnalyser*, https://github.com/OljaOctagon/PoreAnalyser (2023-2030).

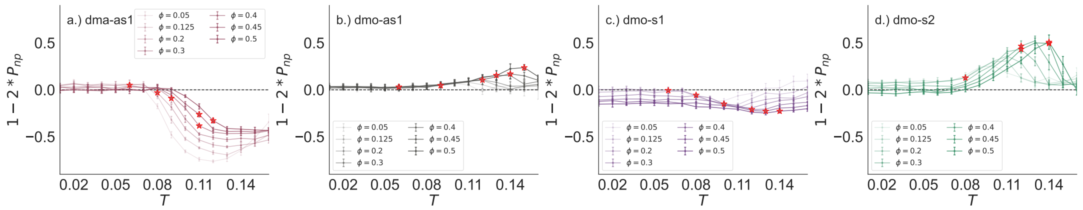


FIG. 1: Bond orientation order parameter $1 - 2 * P_{np}$ as function of temperature $T$, at different packing fractions $\phi = [0.05, 0.125, 0.2, 0.3, 0.4, 0.45, 0.5]$ for particle topolgies with patch position $\Delta = 0.3$: top-bottom Panels (a) dma-as1 (burgundi), (b) dmo-as1 (grey), (c) dmo-s1 (lilac) and (d) dmo-s2 (green), as labelled. Red stars highlight the state point at which percolation occurs.

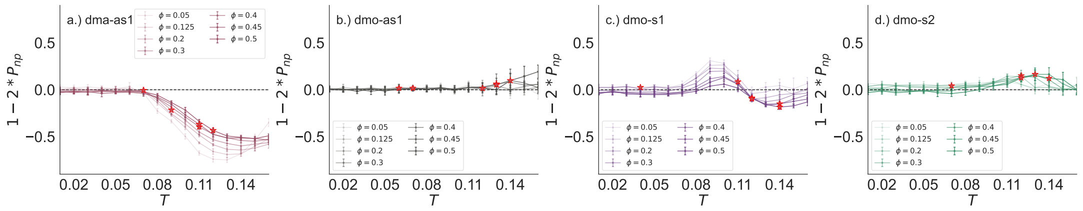


FIG. 2: Bond orientation order parameter $1 - 2 * P_{np}$ as function of temperature $T$, at different packing fractions $\phi = [0.05, 0.125, 0.2, 0.3, 0.4, 0.45, 0.5]$ for particle topolgies with patch position $\Delta = 0.4$: top-bottom Panels (a) dma-as1 (burgundi), (b) dmo-as1 (grey), (c) dmo-s1 (lilac) and (d) dmo-s2 (green), as labelled. Red stars highlight the state point at which percolation occurs.

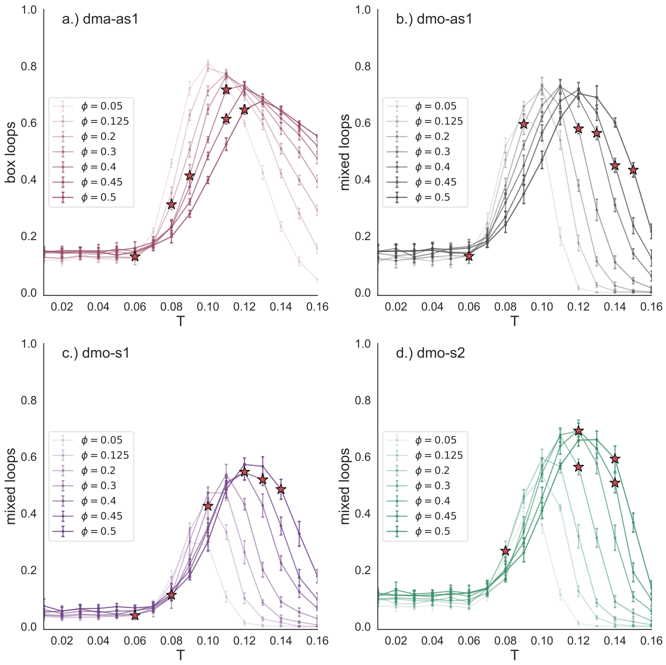


FIG. 3: Fraction of dominant loops type for each particle type as function of temperature T, at patch position $\Delta = 0.3$ and packing fraction $\phi = 0.2$. Panel (a): fraction of dma-as1 particles bonded in open box loops. Panel (b): fraction of dmo-as1 particles bonded in parallel and mixed (parallel and non-parallel) 6-particle loops. Panel (c): fraction of dmo-s1 particles bonded in non-parallel 5- and 6-particle loops. Panel (d): fraction of dmo-s2 particles bonded in parallel and mixed (parallel and non-parallel) 6-particle loops. Red stars highlight the state point at which percolation occurs.

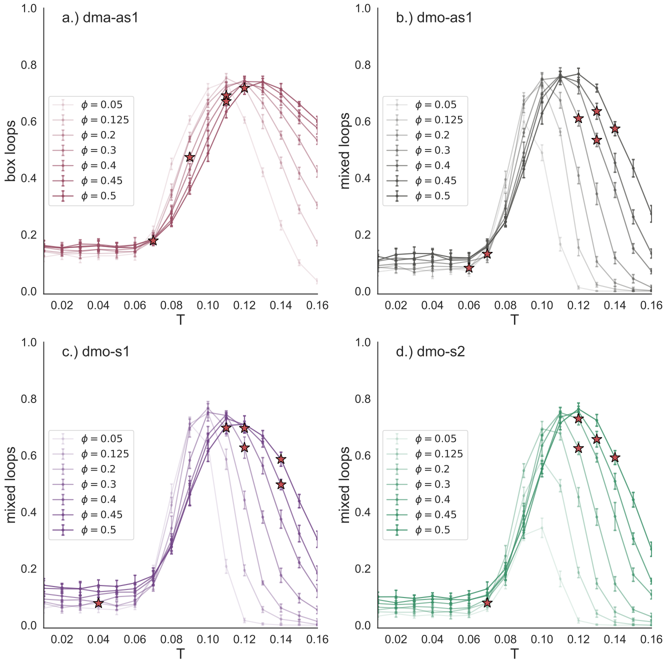


FIG. 4: Fraction of dominant loops type for each particle type as function of temperature T, at patch position $\Delta = 0.4$ and packing fraction $\phi = 0.2$. Panel (a): fraction of dma-as1 particles bonded in open box loops. Panel (b): fraction of dmo-as1 particles bonded in parallel and mixed (parallel and non-parallel) 6-particle loops. Panel (c): fraction of dmo-s1 particles bonded in non-parallel 5- and 6-particle loops. Panel (d): fraction of dmo-s2 particles bonded in parallel and mixed (parallel and non-parallel) 6-particle loops. Red stars highlight the state point at which percolation occurs.

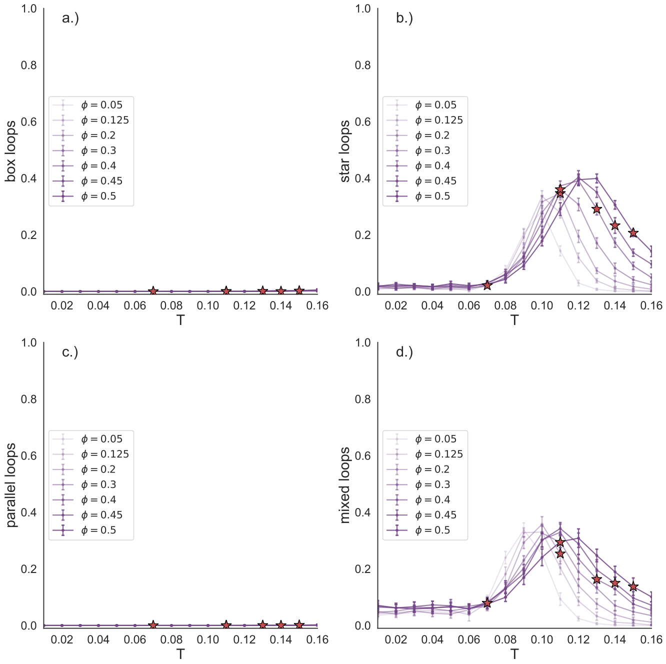


FIG. 5: Fraction of different loops types for dmo-s1 as function of temperature T, at patch position $\Delta = 0.2$ and packing fraction $\phi = 0.2$. Panel (a): fraction of dmo-s1 particles bonded in open box loops; Panel (b): fraction of dmo-s1 particles bonded non-parallel 5- and 6-particle loops (stars); Panel (c): fraction of dmo-s1 particles bonded in parallel 6-particle loops. Panel (d): fraction of dmo-s1 particles bonded in parallel and mixed (parallel and non-parallel) 6-particle loops. Red stars highlight the state point at which percolation occurs.

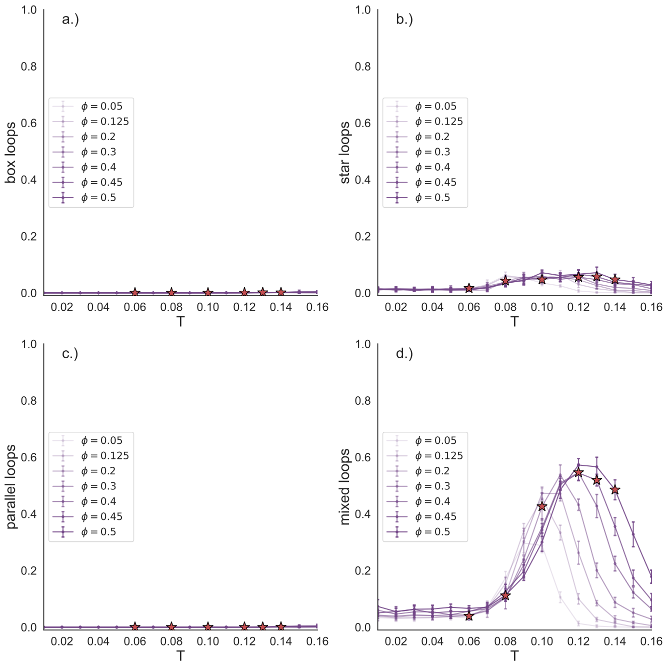


FIG. 6: Fraction of different loops types for dmo-s1 as function of temperature T, at patch position $\Delta = 0.3$ and packing fraction $\phi = 0.2$. Panel (a): fraction of dmo-s1 particles bonded in open box loops; Panel (b): fraction of dmo-s1 particles bonded non-parallel 5- and 6-particle loops (stars); Panel (c): fraction of dmo-s1 particles bonded in parallel 6-particle loops. Panel (d): fraction of dmo-s1 particles bonded in parallel and mixed (parallel and non-parallel) 6-particle loops. Red stars highlight the state point at which percolation occurs.

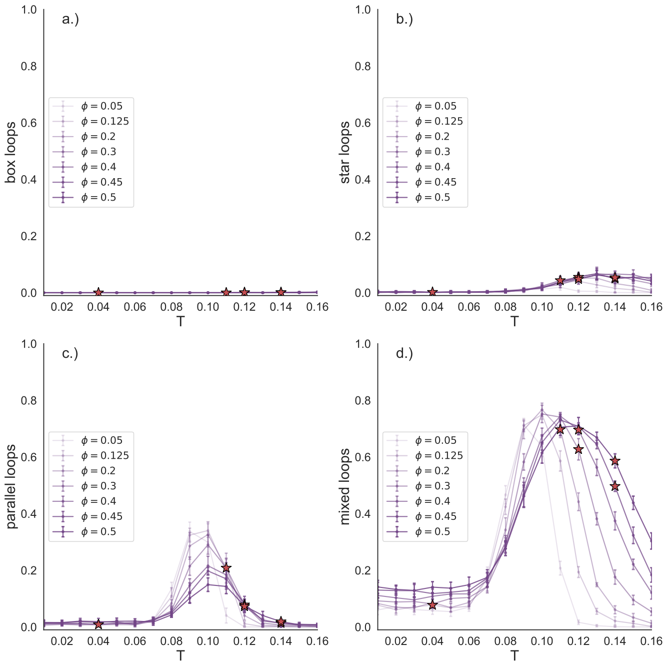


FIG. 7: Fraction of different loops types for dmo-s1 as function of temperature T, at patch position $\Delta = 0.4$ and packing fraction $\phi = 0.2$. Panel (a): fraction of dmo-s1 particles bonded in open box loops; Panel (b): fraction of dmo-s1 particles bonded non-parallel 5- and 6-particle loops (stars); Panel (c): fraction of dmo-s1 particles bonded in parallel 6-particle loops. Panel (d): fraction of dmo-s1 particles bonded in parallel and mixed (parallel and non-parallel) 6-particle loops. Red stars highlight the state point at which percolation occurs.

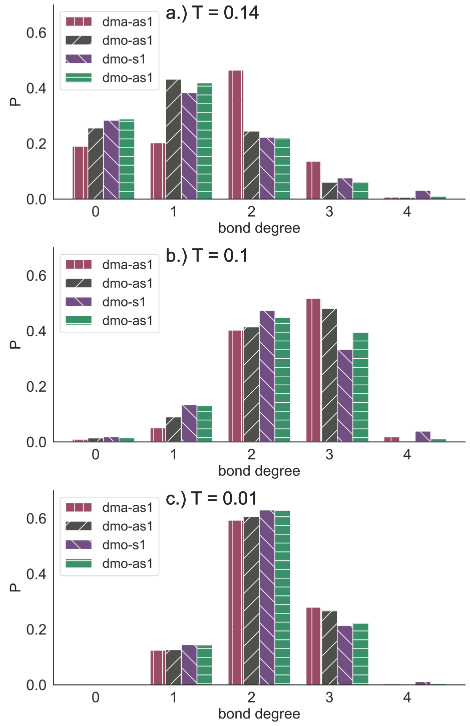


FIG. 8: Bond degree histogram for all particle types -dma-as1 (burgundi), dmo-as1 (gray), dmo-s1 (lilac) and dmo-s2 (green) (as labelled) with patch position $\Delta = 0.3$ and packing fraction $\phi = 0.2$ for (a): temperature $T = 0.14$, (b) temperature $T = 0.10$ and (c) temperature $T = 0.01$.

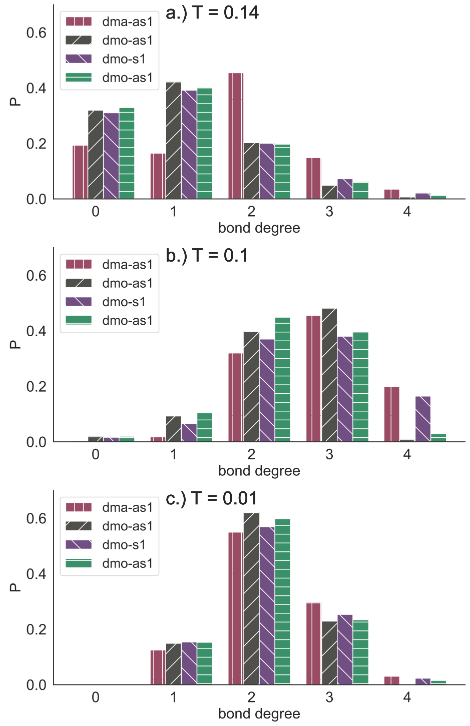


FIG. 9: Bond degree histogram for all particle types -dma-as1 (burgundi), dmo-as1 (gray), dmo-s1 (lilac) and dmo-s2 (green) (as labelled) with patch position $\Delta = 0.4$ and packing fraction $\phi = 0.2$ for (a): temperature $T = 0.14$, (b) temperature $T = 0.10$ and (c) temperature $T = 0.01$.

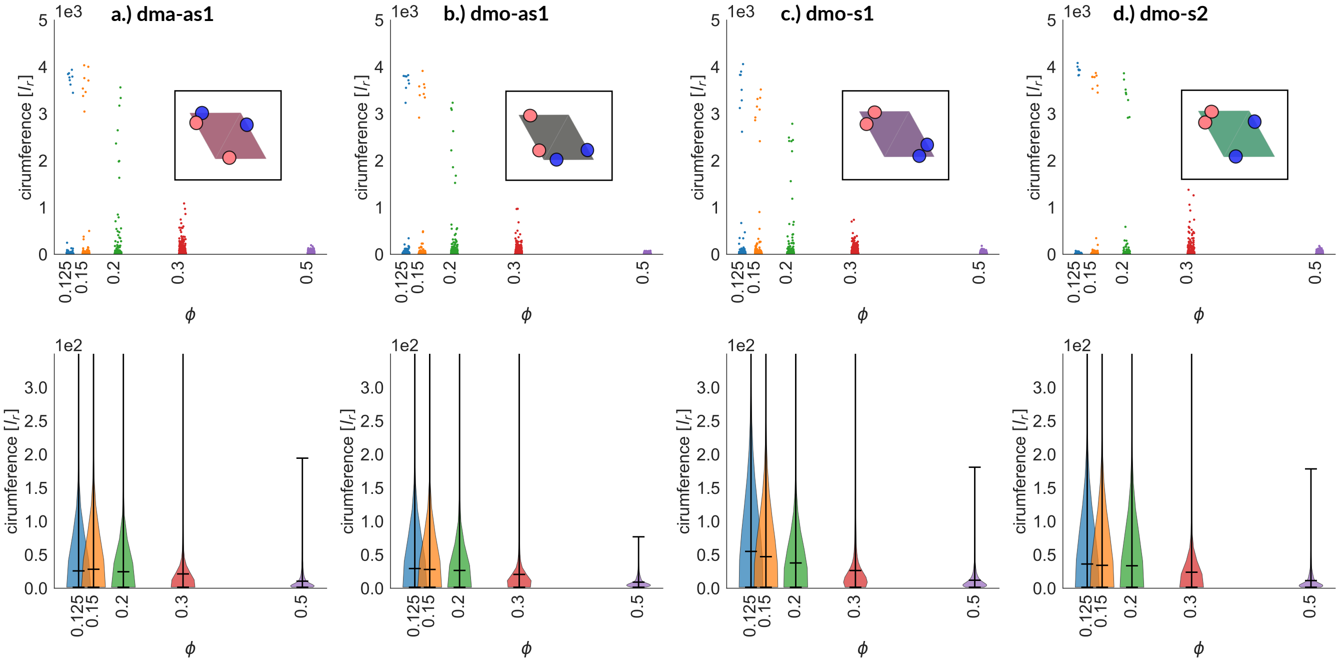


FIG. 10: Pore circumference scatter plots (top row) and pore circumference kernel-density plots (bottom row) for all particle types at patch position $\Delta = 0.2$ and packing fractions $\phi = [0.125, 0.15, 0.2, 0.3, 0.5]$. Panel (a): dma-as1; Panel (b): dmo-as1; Panel (c): dmo-s1; Panel (d): dmo-s2. For better visibility of the scatter, we added random noise to each scatter point in x-direction.

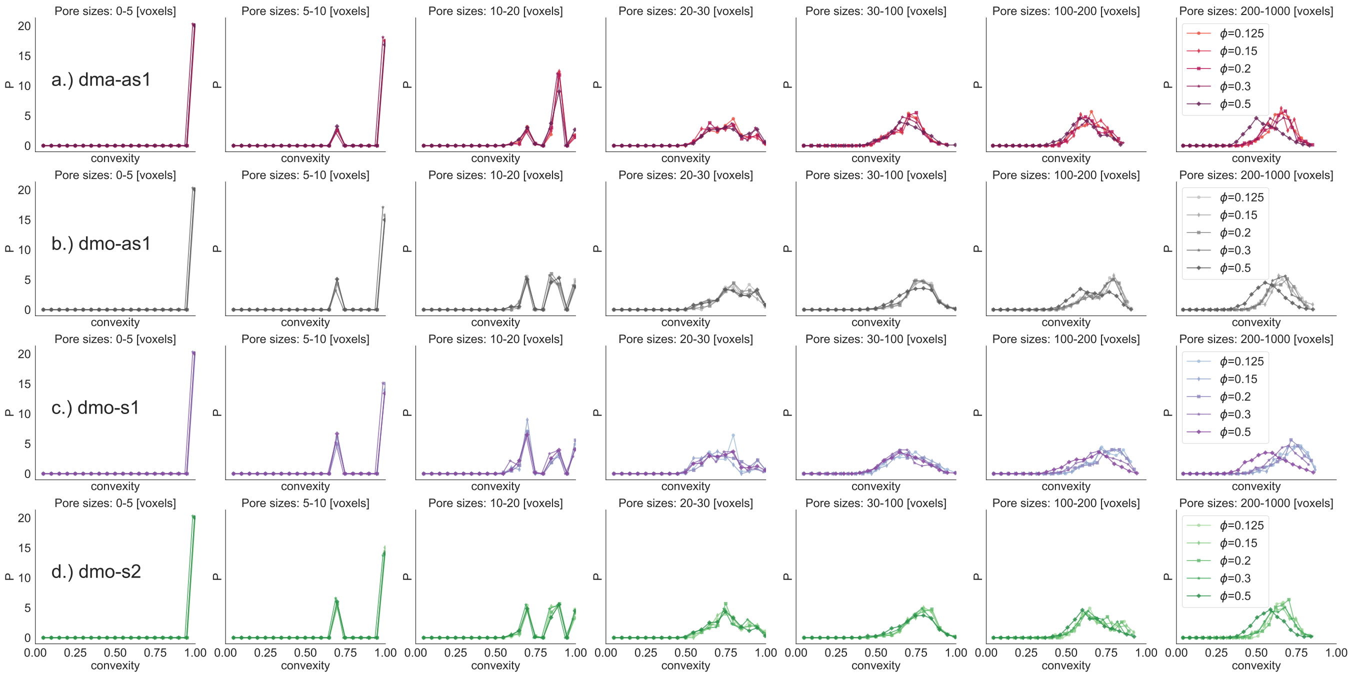


FIG. 11: Convexity measure for different pore size ranges at patch position $\Delta = 0.2$, temperature $T = 0.01$ and packing fractions $\phi = [0.125, 0.15, 0.2, 0.3, 0.4, 0.5]$ for each particle type: Panel (a): dma-as1; Panel (b): dmo-as1; Panel (c): dmo-s1 (c); Panel (d) dmo-s2. Each Panel contains seven plots of the convexity measure with pore sizes increasing from left to right:: pore sizes (0-5), pore sizes (5,10), pore sizes (10-20), pore sizes (20-30), pore sizes (30-100), pore sizes (100-200), pore sizes (200-1000). Note that in this case the pore size is given as number of voxels associated with that pore.

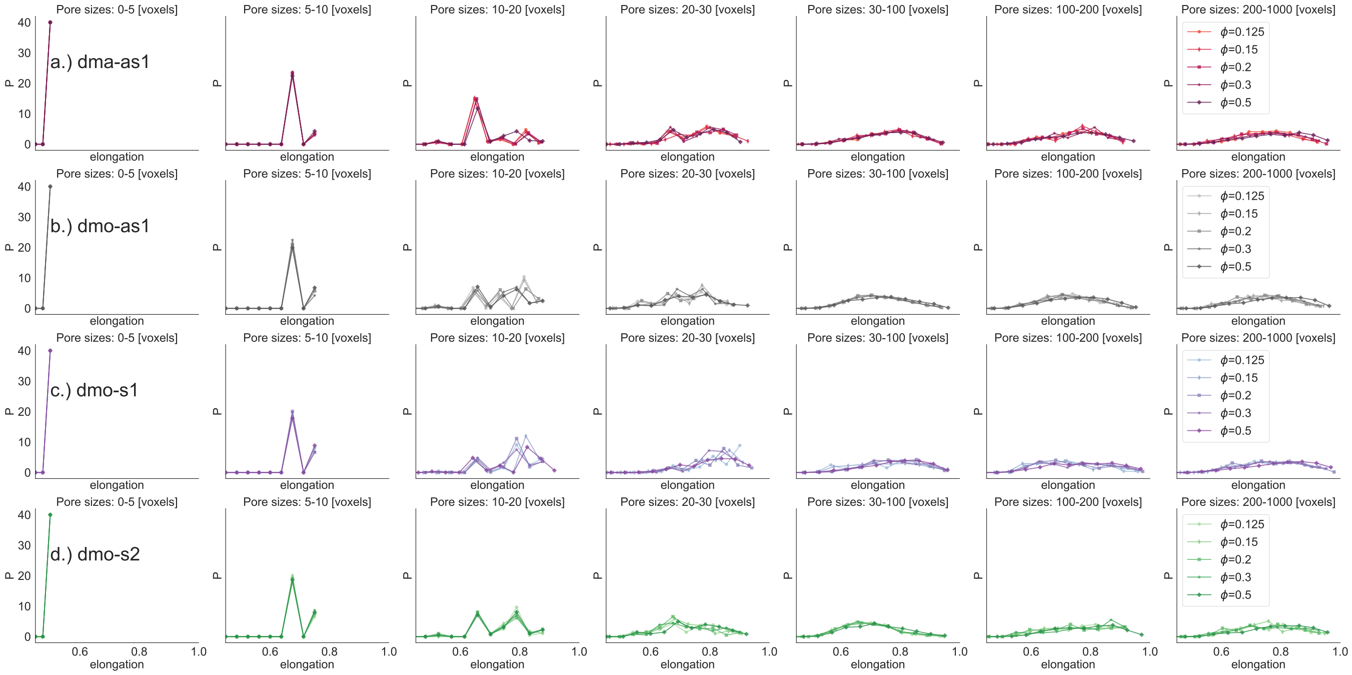


FIG. 12: Elongation measure for different pore size ranges at patch position $\Delta = 0.2$, temperature $T = 0.01$ and packing fractions $\phi = [0.125, 0.15, 0.2, 0.3, 0.4, 0.5]$ for each particle type: Panel (a): dma-as1; Panel (b): dmo-as1; Panel (c): dmo-s1 (c); Panel (d) dmo-s2. Each Panel contains seven plots of the elongation measure with pore sizes increasing from left to right: pore sizes (0-5), pore sizes (5,10), pore sizes (10-20), pore sizes (20-30), pore sizes (30-100), pore sizes (100-200), pore sizes (200-1000). Note that in this case the pore size is given as number of voxels associated with that pore.


[4] B. Barber, *Qhull*, http://www.qhull.org/ (1995–2020).

[5] P. Virtanen, R. Gommers, T. E. Oliphant, M. Haberland, T. Reddy, D. Cournapeau, E. Burovski, P. Peterson, W. Weckesser, J. Bright, et al., Nature methods **17**, 261 (2020).

[6] F. Pedregosa, G. Varoquaux, A. Gramfort, V. Michel, B. Thirion, O. Grisel, M. Blondel, P. Prettenhofer, R. Weiss, V. Dubourg, et al., Journal of Machine Learning Research **12**, 2825 (2011).
